# Place cells in CA1 lack topographical organization of firing locations

**DOI:** 10.1073/pnas.2528601123

**Published:** 2026-02-18

**Authors:** Torstein Slettmoen, Nienke L. de Jong, Hanna Eneqvist, Emilie R. Skytøen, Weijian Zong, May-Britt Moser, Edvard I. Moser

**Affiliations:** ^a^Kavli Institute for Systems Neuroscience and Centre for Algorithms in the Cortex, Norwegian University of Science and Technology, Trondheim 7491, Norway

**Keywords:** hippocampus, place cells, topography

## Abstract

Topography is a well-described and well-known concept for cortical organization in primary sensory and motor cortices of mammalian brains. Similar isomorphic mapping of the environment is absent in most higher association areas such as the hippocampus; however, some studies have suggested that in hippocampal regions spatially tuned cells may be organized in small anatomical clusters with similar firing field locations. Here, we take advantage of the high spatial resolution provided by two-photon calcium imaging to probe the extent of local functional clustering of hippocampal place cells in freely moving mice. Almost one thousand highly packed CA1 neurons were recorded simultaneously per mouse while they foraged freely in open arenas. Among pairs of place cells, there was no relation between preferred firing position and anatomical proximity. Firing fields were no more similar for nearest neighbors than distant cell pairs. When place cells remapped between different arenas, there was no tendency for correlations in one environment to be transferred to the other. A similar lack of relationship between function and anatomical position was expressed in CA1 cells with firing fields tuned to local objects in the environment. The findings suggest that the encoding of location in CA1 place cells is likely fully nontopographical.

## Significance

Unlike neurons in sensory and motor cortices, hippocampal place cells do not exhibit large-scale topographic organization. Some studies have suggested, however, that amid a distributed organization, hippocampal neurons assemble into small clusters with correlated properties. Here, we used a miniaturized two-photon microscope to elucidate at high spatial resolution whether such clustering exists in CA1 place cells of freely moving mice. Recording from almost a thousand neurons per trial, we found no evidence for clustering, despite the increase in anatomical resolution and statistical power compared to previous approaches. A fully distributed organization is consistent with the ability of CA1 place cells to express large numbers of orthogonal position maps and the participation of such maps in high-capacity memory storage.

The nervous system is thought to express properties of the world in activity patterns of neural ensembles, recreating features of the surroundings in “neural representations.” Such representations are often expressed in a topographic organization of functional cell types in the sense that cells are anatomically arranged according to distance between their receptive fields in the external world. However, while these isomorphic maps are prominent in the primary sensory and motor cortices of many mammals (1–12), higher association cortices are organized differently, often with similarly tuned neurons distributed widely within the circuit. Such distributed networks could take the form of small-world networks with local connections between similar neurons (13–15) or function could be fully distributed in a salt-and-pepper fashion as observed in some sensory cortices of the rodent brain (16, 17). In hippocampal and parahippocampal areas, topographical organization is present in the sense that spatially tuned neurons gradually increase their scale along the dorsoventral axis (18–21), but it was apparent already from early work on spatially tuned cells that there is no large-scale isomorphism between the location of firing fields and the location of neurons in the circuit, neither in place cells of the hippocampus (22–24) nor in grid cells of the medial entorhinal cortex (19).

Later work has pointed to the possibility that within a largely nontopographic place cell or grid cell map, there may be small-scale organization in the form of local cell clusters with more similar spatial tuning than expected by chance (25–28) or with a common tuning to task variables or movement parameters such as speed, direction, and turning angle (25, 27). The suggested clustering of place cells would be consistent with a small-world network organization (13, 14) but the validity of the proposal remained unsettled due to low cell yields and low anatomical precision of the methods used at the time, most being electrophysiological recordings. A game changer was the introduction of calcium imaging to image hippocampal neural activity. With the increased spatial resolution of this method, studies confirmed that CA1 lacks a large-scale topographic mapping of the spatial environment. However, some investigations noticed the presence of local clusters of place cells with correlated properties, mirroring claims of small-scale organization from the early electrophysiology studies. In one study, the authors used one-photon (1P) calcium imaging in freely moving rats to identify clustering of place fields with common firing fields at past reward locations (29). In another study, using higher-resolution two-photon (2P) calcium imaging during running in head-fixed mice, the authors noticed increased similarity of firing field locations among nearest neighbors of CA1 place cells when the distance between the cells was smaller than the width of 1 to 2 pyramidal cell somata (30). A similar nearest-neighbor correlation was reported with 2P imaging in grid cells of the MEC (31). Taken together, these studies introduced cellular resolution to the study of topography in spatially tuned neurons but as noted by some of the authors, the small correlations observed between nearest neighbors of place cells could also reflect limitations of the methods of the time, such as bleed-through of signal from neighboring neurons (30). Moreover, while 2P imaging provides better resolution and less signal contamination than 1P studies, 2P studies have until recently required animals to be head-fixed under a benchtop system. The latter experiments have enforced navigation to take place in a virtual reality environment, at the risk of impairing the animals’ naturalistic positional computation and spatial map formation (32, 33).

In the present study, we took advantage of recent methodological advances to revisit the question of small-scale topographical organization in local ensembles of place cells. First, we employed transgenic mice expressing a more sensitive fluorescent indicator—GCaMP6 (34)—than in the early 2P studies of place cell topography (30). The transgenic line ensured expression in most excitatory neurons, enabling the vast majority of cells in the field of view (FOV) to be included in the dataset. Second, we utilized a miniaturized 2P microscope (MINI2P) (35) to simultaneously record activity from many hundreds, and in some cases nearly a thousand, CA1 neurons, all while the mice moved freely in two-dimensional open environments, without the vestibular constraints of head-fixation. Third, to maximize the quality of recordings from the hippocampus, we optimized the optical window by using a customized thick-glass implant, referred to as a glass plug, compatible with MINI2P (36, 37). This offered higher resolution than previously reported GRIN lenses without reducing the size of the FOV (35), and also resulted in less cortical damage than in prior 2P studies (30). With the marked improvement in throughput and resolution resulting from these advances, we were able to comprehensively reinvestigate the functional similarity of neighboring place cells in CA1 of the hippocampus. Our findings strongly indicate that neuronal representations of place in the CA1 region are entirely nontopographical, with no additional similarity in spatial firing properties among nearest neighbors compared to cells further apart.

## Results

**Fig. 1.**
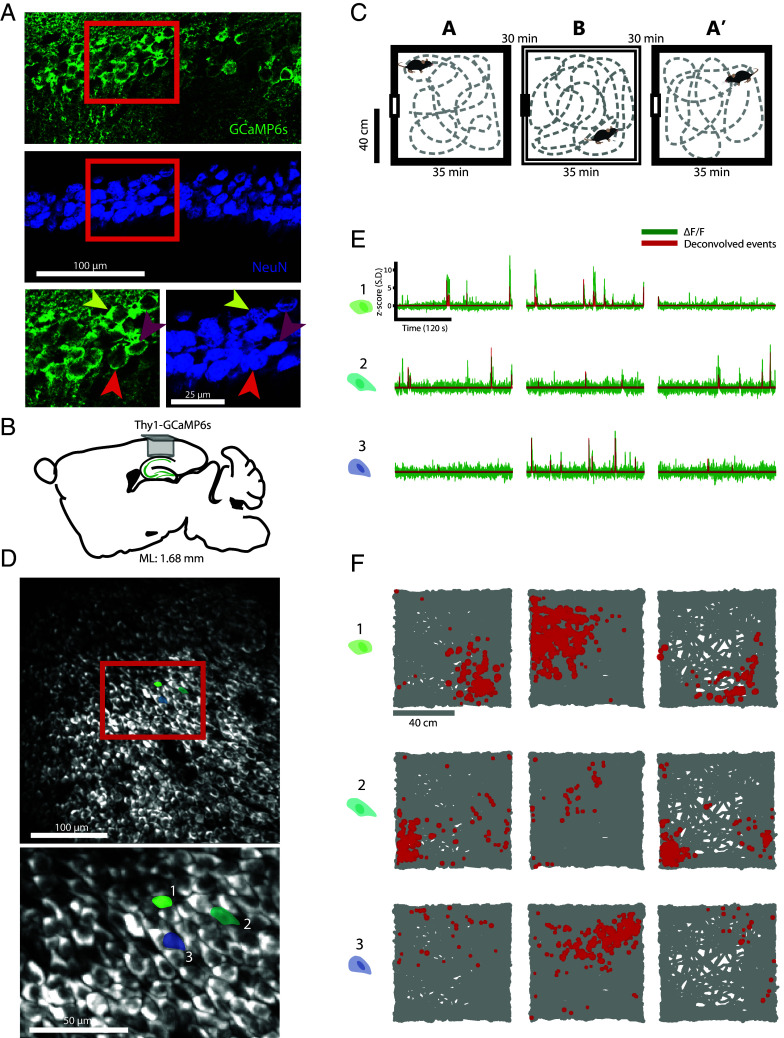
MINI2P allows recordings of hundreds to a thousand excitatory CA1 cells. (*A*) Expression in transgenic Thy1-GCaMP6s mice. Confocal image from a sagittal section of CA1 showing the expression of neuronal marker NeuN (blue) and GCaMP6s (green). The highlighted area shows that most NeuN+ cells express GCaMP6s, exemplified by the three arrows indicating three neurons coexpressing NeuN and GCaMP6s. (*B*) Schematic illustrating optical access to CA1. Adult Thy1-GC6s mice underwent surgery in which the cortex above the alveus was carefully aspirated and a glass plug (diameter: 1.8 mm; length: 1.5 mm) was implanted above CA1. The mouse line expresses GCaMP6s in excitatory neurons of the cortex, here depicted by green shading only for hippocampal cell layers of CA1-3 and dentate gyrus. ML: Medio-lateral. (*C*) Experimental protocol. Neural activity was recorded with the miniature two-photon microscope MINI2P while mice foraged successively in two different open field environments in an A-B-A’ remapping protocol. (*D*) Example imaging field of view (FOV). Up to one thousand excitatory CA1 cells could be distinguished during imaging in one FOV (*Upper* panel). *Lower* panel shows zoomed-in image from the FOV (corresponding to the red enclosed area in the *Upper* panel). Three place cells are marked out with the cell outline (light color: cytoplasm; dark color: nucleus); activity of those cells is shown in panels *E* and *F*. (*E*) Single cell traces. Activity of single cells was measured as change in fluorescent GCaMP6s-signal. From the raw ∆F/F (depicted as green traces, z-scored SD), cell activity was approximated by deconvolution of the calcium trace to deconvolved events (depicted as red traces, z-scored). The three rows correspond to the three cells marked out in *D*, and the columns correspond to the environments (box A-B-A’) in which the recording was done. (*F*) Place cells in CA1. The trajectory of the mouse is plotted in gray, superimposed with the cell’s deconvolved calcium events (red dots). The size of the dots corresponds to the amplitude of the deconvolved signal. Rows correspond to the cells depicted in *D*, and columns to the environments A-B-A’, respectively.

Thy1-GCaMP6s mice expressing GCaMP6s in excitatory neurons of the hippocampus (38) were imaged using a MINI2P microscope (35) targeting the CA1 area of the hippocampus. To first assess if the expression of GCaMP in this mouse line was consistent across the CA1 area, we manually counted, in confocal microscopic images, the number of cells within CA1 expressing the immunohistochemical neuronal marker NeuN as well as the number of NeuN cells coexpressing the green fluorescent protein (GFP). Cell counts were stable across three mice (three sections per mouse at corresponding locations; see *Materials and Methods* for details), as was the proportions of GFP+ cells in these sections (mean proportion of GFP+ cells ± SD per mouse: 82.64 ± 2.10%, 80.75 ± 1.76%, 84.68 ± 1.45%; Fig. 1*A*). In total across all sections, 3,428 cells were NeuN+, of which 2,831 cells (82.58%) were GFP+, suggesting high and consistent expression of GCaMP6s in CA1.

For optical access to the CA1 cell layer, we implanted a glass plug (diameter 1.8 mm, length 1.5 mm), glued to a coverslip, above dorsal CA1 and at the bottom of a narrow tunnel of aspirated cortex (Fig. 1*B*). Compared to GRIN lenses, imaging through a glass plug causes less optical aberration and higher spatial resolution. To record place cells, we let the mice explore freely in two open field environments (A and B, both 80 cm × 80 cm), with two trials in the A environment (A-B-A’ sequence) (Fig. 1*C*). MINI2P allowed us to record place cell dynamics with high resolution in a FOV of approximately 350 µm × 350 µm (Fig. 1*D*). Due to the high spatial resolution of MINI2P, combined with a robust signal extraction algorithm (39), we could consistently separate nearest-neighbor cells and follow their distinct fluorescence patterns with minimal contamination from neighboring and out-of-plane cells (35) (Fig. 1 *D*–*F*).

Across five mice, we imaged a total number of 8,762 cells, of which 7,983 (91%) had a signal-to-noise ratio (SNR) > 3 (mean SNR ± SD: 5.39 ± 2.00), indicating an overall robustness in cell separation and signal extraction. At most, 1,000 cells were recorded within a single FOV (from 358 to 1,000 cells per FOV across all experiments). In two of the animals, different FOVs were recorded in the same CA1 subfield by shifting MINI2P in the anteroposterior and mediolateral directions using stitching adapters (35). With this procedure we obtained 4,163 and 2,940 cells from the two animals, respectively. The other three mice yielded 358, 490, and 811 cells. Of all recorded excitatory cells in CA1, a total of 6,519 (74%, ranging from 45 to 85% between experiments) were classified as place cells, with fields in either one or both environments (A or B), based on the spatial information content of their activity, the stability of their activity within a session, and the existence of one or several localized place fields (*Materials and Methods* and *SI Appendix*, Fig. S1). Within each of the 80 cm × 80 cm environments, the estimated percentage of active place cells was 35 to 40%, and in the range of previous estimates of the fraction of active place cells in similarly sized arenas in the dorsal CA1 field (40, 41). The position of the implant and the expression of GCaMP6s were verified by histological staining in all animals (*SI Appendix*, Fig. S2).

### Pairwise Comparison of Place Cells Confirms Lack of Global Topography.

**Fig. 2.**
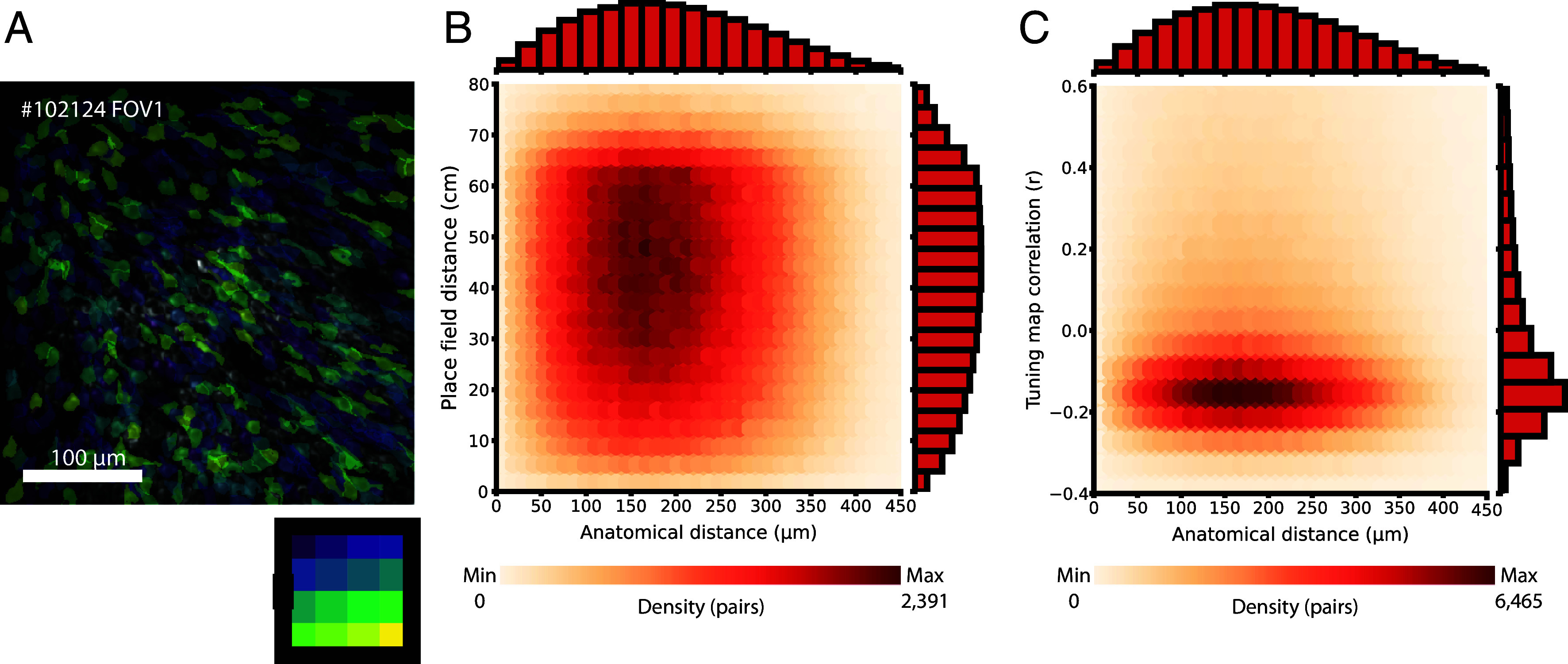
Pairwise place cell relations do not reveal a topographic distribution. (*A*) Place cells are scattered around the FOV. For the same FOV as in Fig. 1*D*, all place cells in environment A are color-coded by where in the open field the centroid of their place field was located. Location in the environment is indicated in the color-matrix at the *Bottom Right* (each color covers a 20 cm × 20 cm square of the environment). For all FOVs from all animals, see *SI Appendix*, Fig. S2. (*B*) Anatomical distance and place field show no clear relationship. Heat map showing the relationship between anatomical distance (μm) and place field distance (cm) for all sets of place cell pairs for all sessions. Data were pooled across environments *A* and *B* (A’ was left out; n = 1,731,288 place cell pairs). Scale bar indicates density of datapoints per bin. Distribution histograms for anatomical distance and place-field distance are added to the *Top* and *Right* side, respectively. (*C*) Anatomical distance and rate map show no clear relationship. Similar to *B*, but instead showing anatomical distance as a function of correlation between spatial tuning maps of place cell pairs. There was a tendency across animals and FOVs for a slight negative correlation between spatial tuning maps of place cell pairs, likely reflecting that place cell maps have a spatial confined zone and that two place cells will tend to have different activity positions in the box, resulting in being slightly negatively correlated.

First, to rule out any possible large-scale topography in place fields of place cells, we identified all place cells from a single environment in the imaging FOV. The color code in Fig. 2*A* indicates where in the recording arena the cells’ place fields were located (see also *SI Appendix*, Fig. S3). To quantify the extent of topography, we calculated the correlation between two place cells’ anatomical distance with either their place field distance (Fig. 2*B*) or the correlation of their spatial tuning maps (Fig. 2*C*) for all cell pairs.

In the full sample of place cells (n = 1.7 million cell pairs), there was no significant relation between the anatomical distance and the place field distance of the cell pairs (Fig. 2*B*), and the mutual information (MI) for the pair of continuous variables was low (MI = 0.00019). Likewise, there was no significant relation between the cell pairs’ anatomical distance and the correlation of their spatial tuning maps (Fig. 2*C*; MI = 0.00024). Similar results were obtained when sessions were analyzed separately (*SI Appendix*, Fig. S4).

### Absence of Functional Clustering in Nearby Place Cells.

**Fig. 3.**
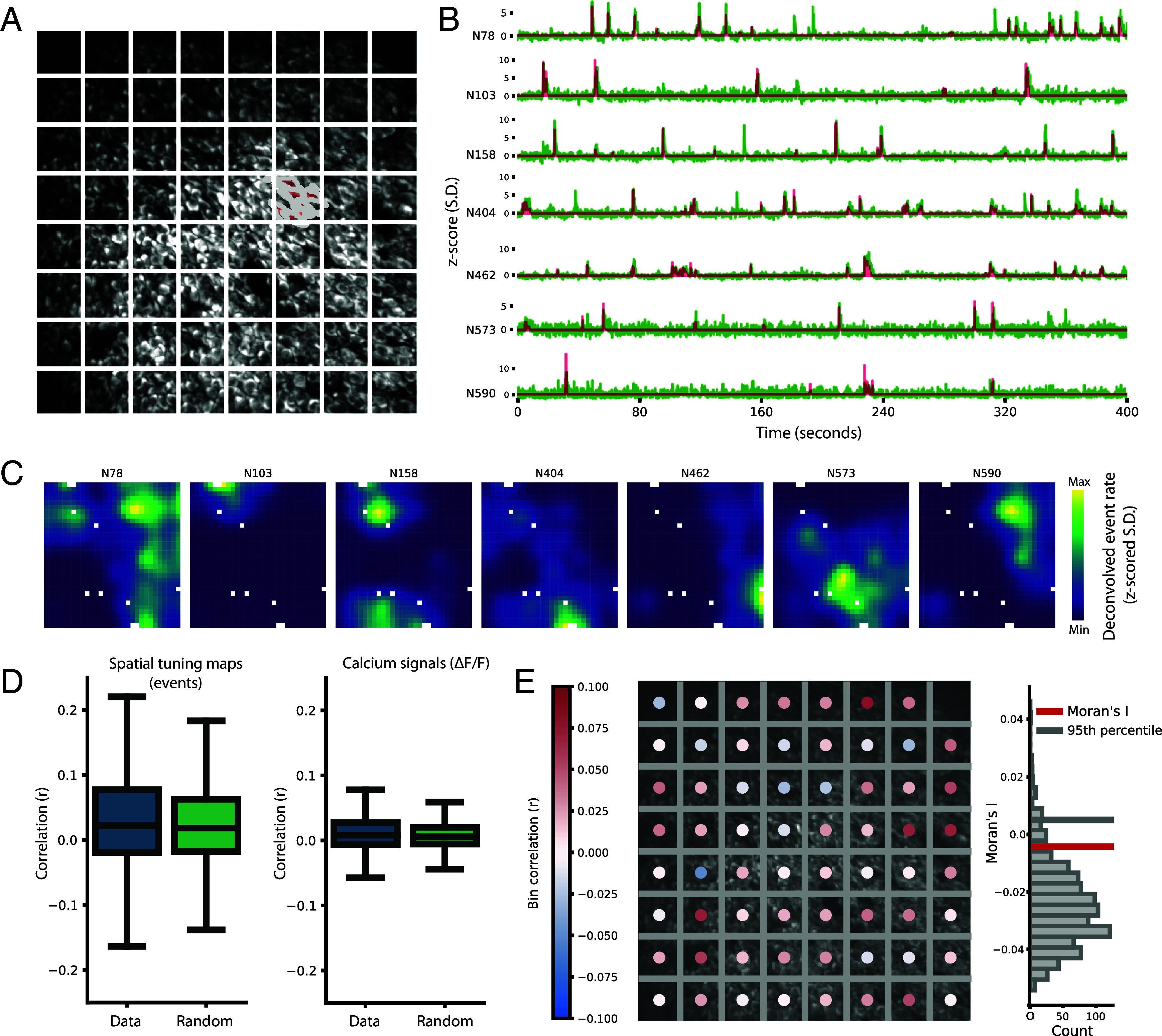
Lack of topographic distribution for binned nearby place cells. (*A*) Anatomical binning of place cells. Maximum intensity projection from one FOV. The cells were assigned to bins of approximately 45 µm × 45 µm (white squares). ROIs of seven example place cells belonging to one bin are outlined and filled in red. (*B*) Signal traces of the place cells shown in red in A, all from the same bin. For each place cell, the calcium signal from 400 s in environment *A* is shown. Green traces are z-scored ∆F/F; red traces are z-scored deconvolved events. (*C*) Spatial tuning maps of place cells. Spatial tuning maps of the place cells shown in *A* and *B*. Lighter colors indicate higher rate of deconvolved calcium events (z-scored; color bar to the *Right*). The 12 spatial bins which the animal did not visit are displayed in white. (*D*) Spatial correlation of place cell activity among pairs of cells belonging to the same anatomical bin is not different from randomly picked samples. Box plot showing median correlation values (horizontal line) with interquartile ranges (IQR, box) and whiskers at 1.5 times the IQR for recorded data and randomly picked samples. The random control was size-matched and drawn from the full population of place cells for that session. For all sessions and environments, only anatomical bins with at least 3 place cells were analyzed. Place cell activity of recorded data and random control was compared in two ways: *Left* panel: Pearson correlation of the spatial tuning maps of deconvolved event rates for all place cells within one anatomical bin compared to that of a size-matched sample of randomly chosen place cells. *Right* panel: Pearson correlation of ∆F/F for all place cells within the same anatomical bin compared to a size-matched sample of randomly chosen place cells. (*E*) Moran’s I—a measure of spatial clustering—was not different from that of randomly picked samples *Left*: Same FOV as in the example session in *A*, with superimposed dots color coded by the mean of the pairwise Pearson correlation of spatial tuning maps for the place cells within the bins (as in *D*). Red color indicates positive correlation, while blue color indicates negative correlation. *Right*: From this example session, the global Moran’s I was calculated for the distribution of correlation values across bins. The histogram shows the distribution of the random control data with the measured Moran’s I (red line) and the 95th percentile of the random distribution (gray line).

In the absence of evidence for a global topographical distribution, we wondered if topography was expressed locally in domains of nearby place cells, as reported in some former studies (25–30). First, we binned the FOV into equally sized bins of approximately 45 µm × 45 µm and grouped place cells together if they were in the same anatomical bin (Fig. 3*A*). The bin size was chosen based on a previous report of possible local topography within such distances (30) and our desire to keep both a high number of bins (n = 1,176 bins with place cells across all sessions) and a reasonable number of place cells per bin (mean number of place cells in all bins ± SD: 7.0 ± 2.7 place cells). Only bins with at least three place cells were considered. For each place cell, we determined the calcium activity (ΔF/F and deconvolved events) (Fig. 3*B*) and calculated the cell’s spatial tuning map (Fig. 3*C*). We then calculated the mean correlation of spatial tuning maps between pairs of place cells within an anatomical bin (not including pairs closer than 15 µm due to overlap among neighboring cells), and we then asked if those place cells had higher correlation than a size-matched control of randomly chosen place cells from the same session. The median spatial tuning map correlations between place cell pairs within bins were not different from that of the randomly chosen sample [Fig. 3*D*; median of spatial tuning map correlation within bins with 99% CI from bootstrapping with 10,000 resamples: 0.0234 (0.0172, 0.0303); median of the control sample, 200 iterations per bin: 0.0184]. We also directly correlated the calcium signal (ΔF/F) of place cells within anatomical bins (Fig. 3*B*) and compared the results to size-matched controls of randomly chosen place cells from the same session (Fig. 3*D*). Again, the correlations within bins were not different from those of the size-matched control [median of calcium signal correlation within bins with 99% CI from bootstrapping with 10,000 resamples: 0.00816 (0.00576, 0.0105); median of the control, 200 iterations per bin: 0.00590].

In a second approach to identifying local clustering in the above data, we computed the global Moran’s I, a measure of spatial autocorrelation that quantifies how clustered a one-dimensional variable of interest is (42). This quantification, given by the value of I, considers both the value of the variable and the location of it, and may be used to quantify how clustered or dispersed areas with similar values of the interest variable are (for details, see *Materials and Methods*). Using the mean of spatial tuning map correlations within each anatomical bin as our variable of interest, we calculated the global Moran’s I for all FOVs. We found no indication of clustering (Moran’s I < 95th percentile of a random distribution for all 24 sessions; Fig. 3*E* and *SI Appendix*, Fig. S5). Further, we decomposed the global Moran’s I into local Moran’s I, determined for each anatomical bin, to assess if there were any minor clusters not unraveled by the global Moran’s I. Across all sessions (n = 24), only two had significant local Moran’s I values; however none of these showed evidence of high-correlation clusters (*SI Appendix*, Fig. S6 *A* and *B*). Taken together, the Moran’s I analyses point to a random anatomical distribution of place cells based on their spatial tuning properties, without local clusters of similarly tuned place cells.

**Fig. 4.**
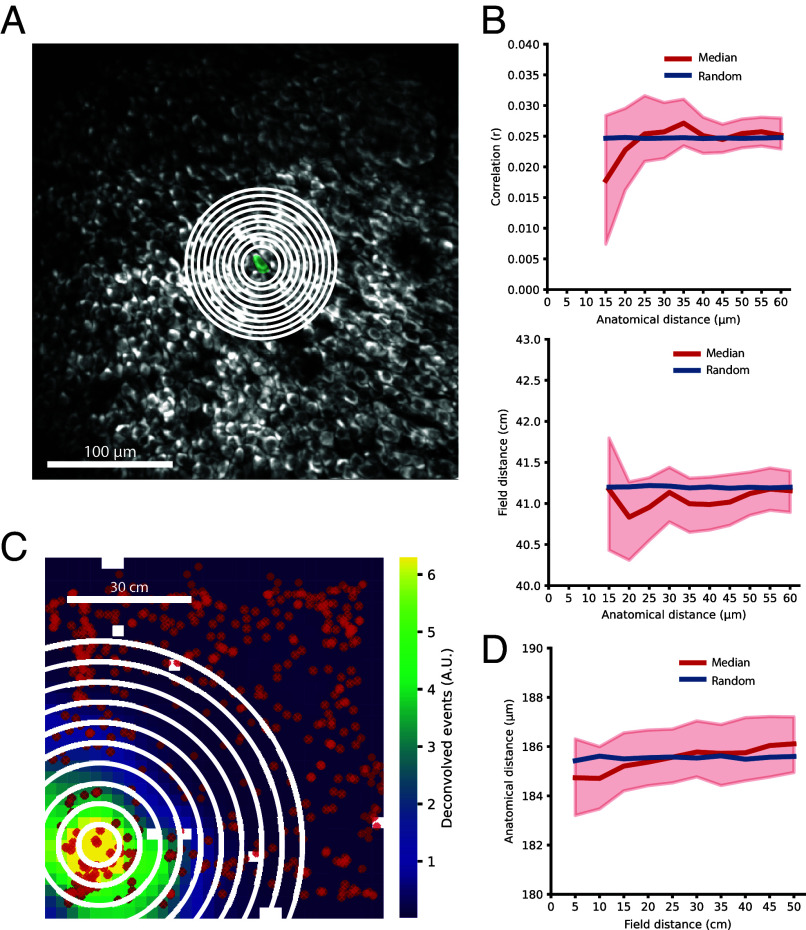
Topographic distribution of incremental nearby place cells. (*A*) Anatomical binning of place cells by an expanding circle. FOV from one session with an example place cell in green surrounded by expanding circles of 5 µm increments starting from a radius of 15 µm. For each single place cell, we identified all neighboring cells within incremental distances (defined by the expanding circles) from the reference place cell’s center. The median of the correlations between the reference place cell’s spatial tuning map and that of each neighboring place cell was calculated. (*B*) Spatial correlation (*Top*) and place field distance (*Bottom*) between place cells as a function of anatomical distance in circular bins. Plot showing the median of either spatial tuning map correlations (*Top*) or place field distances (*Bottom*) at increasing anatomical distance from a reference cell in red with 99% CI (red shaded area) together with the median value of a randomly picked control (blue). (*C*) Binning by an expanding circle over place fields in the arena. Example spatial tuning map from one place cell based on deconvolved events, as in Fig. 3*C*. Red crosses indicate the place field centers of all other place cells in that session. Place fields of neighboring cells were defined by an expanding circle from the center of the place field of the reference cell. The median anatomical distance to all neighboring cells was determined for each circle (see panel *D*). (*D*) Anatomical distance between place cells as a function of place field distance. Similar to panel *B* but showing median anatomical distance as a function of increasing place field distances (red) with 99% CI and the median of a randomly picked control (blue).

In a third approach, we used single place cells in the FOV as the reference, comparing the spatial tuning of each individual cell to that of its local neighbors. Each reference cell was compared with all nearby place cells at incremental anatomical distances from the reference cell (Fig. 4*A*). For each distance step, the Pearson correlation between the cells pairs’ spatial tuning maps as well as their place field distances were calculated, and further the median and the 99% CI (from 10,000 bootstraps) of these correlations and field distances (Fig. 4*B*). These were compared to the median of size-matched randomly chosen distributions of place cells from the same session (n = 200 iterations) taken from calculated correlations at all anatomical distances. At all increments, the observed data and the random control samples were similar, with the random sample falling within the 99% confidence bounds of the observed data. Thus, there was no meaningful change in either tuning map correlation nor place field distance as a function of anatomical distance from the reference cell (Cliff’s delta for effect size between correlation 15 µm and 60 µm: d = −0.023, n = 82,468; Cliff’s delta for effect size between field distance at 15 µm and 60 µm: d = −0.013, n = 82,468).

Next, we flipped the perspective and asked whether we could see anatomical clustering among place cells with nearby place fields in the open field environment. In a similar fashion as in Fig. 4 *A* and *B*, we took the centroid of the place field of one reference place cell and identified place cells with a place field centroid within an incremental distance from the reference place cell’s field (Fig. 4*C*). For each reference place cell, and at each increment of distances, we calculated the median anatomical distance of all pairwise comparisons to the reference cell and its 99% CI (from 10,000 bootstraps). When compared to size-matched randomly chosen controls of cells from all anatomical distances (n = 200 iterations), the random control sample fell within the CI for all field distance increments (Fig. 4*D*). Also, the absolute increase of median distance from the first (5 cm) to the last group (50 cm) was only 1.38 µm (Cliff’s delta for effect size between the place field distances at 5 cm and 50 cm: d = −0.034, n = 84,373), suggesting no meaningful relation between nearby place fields and anatomical proximity.

**Fig. 5.**
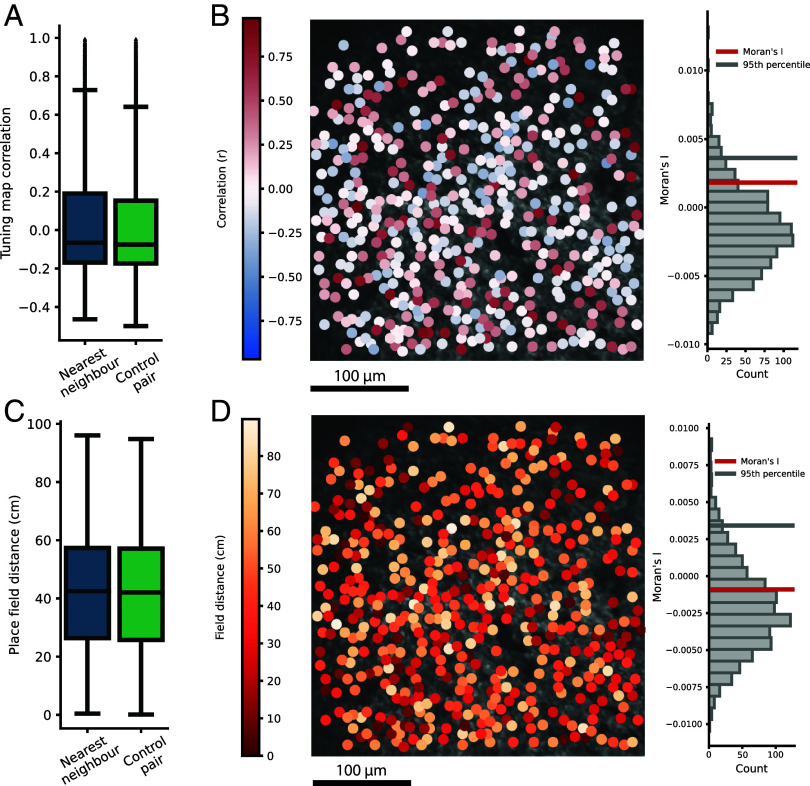
Place cells are not tuned similarly to their nearest neighbors and exhibit no clustering. (*A*) Nearest neighbor place cells do not have similar spatial tuning. Box plot, as in previous figures, showing the correlation of spatial tuning maps between each single place cell and its nearest place cell neighbor (blue) compared to the spatial tuning map correlations for each single place cell with reference to that of a randomly selected place cell from the same session (green). Data from environment *A* and *B* are combined from all 12 experiments (*A* and *B*;*A*’ not included), using all place cells per environment. (*B*) Global Moran’s I shows random distribution of place cells by comparing their tuning to the nearest neighbor. Similarly to Fig. 3*E*, we recomputed the global Moran’s statistics using all place cells (dots) and the cells’ correlation of spatial tuning map to its nearest place cell neighbor (color, Scale bar at Left), distributed across the FOV. The Moran’s statistic, to the *Right*, shows the randomly picked controls (gray histogram) with its 95th percentile (gray vertical line) together with the calculated Moran’s I (red vertical line), which is consistent with a random distribution. (*C*) Nearest neighbor place cells do not have shorter distance between place fields. As in *A*, showing the place field distance between each place cell to its nearest neighbor (blue) compared to that of a random place cell from the same session (green). (*D*) Global Moran’s I shows random distribution of place cells by tuning to the nearest neighbor. The same FOV as in B showing one dot per place cell color-coded by the place field distance to its nearest place cell neighbor (Scale bar at *Left*). As for spatial tuning map correlations, the Moran’s I is consistent with a random distribution rather than clustering.

Finally, we sought to evaluate signs of topography without binning, as binning would not allow the identification of topography at a smaller scale than the bin size and comes with the risk of cutting across possible clusters of similarly tuned place cells, with a consequent failure to discover the clusters. In a bin-free analysis, we compared the spatial tuning of each single place cell to that of its nearest-neighbor place cell, whatever the distance was between those two cells. First, we calculated the correlation between the spatial tuning maps of each pair of closest neighbors and compared it to the spatial tuning map correlations between each place cell and a randomly picked place cell in the same session [Fig. 5*A*; median of spatial tuning map correlation of nearest neighbors with 99% CI from bootstrapping with 10,000 resamples: −0.0234 (−0.1068, −0.0055); median of random control sample: −0.0792]. Similarly, the global Moran’s I revealed no signs of clustering (Fig. 5*B*) (Moran’s I < 95th percentile of a random distribution for all 24 sessions). In a second approach, we calculated the place field distance between each place cell to that of its nearest place cell and compared it to the distance of a randomly picked place cell in the same session (Fig. 5*C*). This again revealed no differences [median place field distance of nearest neighbors with 99% CI from bootstrapping with 10,000 resamples: 42.50 (41.69, 43.32); median of random control sample: 41.97]. The global Moran’s I revealed no signs of clustering for all but one FOV (Fig. 5*D*) (Moran’s I < 95th percentile of a random distribution for 23/24 sessions). However, in the single FOV with significant Moran’s I, decomposing the global Moran’s I into local Moran’s I failed to identify cells with tuning similar to that of its neighbors (*SI Appendix*, Fig. S6 *C* and *D*).

### Relative Firing Positions of Place Cells Are Not Maintained During Remapping.

**Fig. 6.**
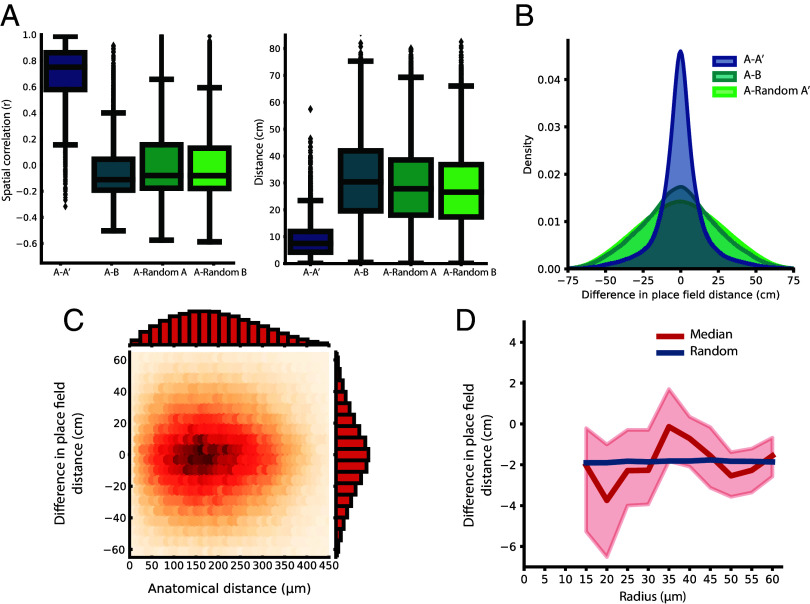
Relations between place cells are lost in remapping. (*A*) Global remapping between environments. Box plot showing that for place cells active in both environments, the correlation between individual cells’ spatial tuning maps (*Left*) and shifts in center of mass (*Right*) in environments A-B are not different from that of randomly picked place cells. Symbols as in Fig. 3*D*. (*B*) Change in place field distance of cell pairs between environments is kept within the same environment but not between environments. Kernel density estimate of the difference between place field distances of cell pairs in the two different environments, across all sessions. The difference in pairwise distance between the main place field within one environment (*A-A*’) was compared to the distance between environments (*A* and *B*) and to a distribution of randomly picked place cells from the same environment (A-Random *A*’). (*C*) Pairwise differences in place field distance from environments *A* to *B*. Heat map as in Fig. 2 *A* and *B*, showing for cell pairs with activity in both environments the relation between difference in pairwise place field distance from A to B and the pairwise anatomical distance. There is no subpopulation of neighboring place cells (small anatomical distance) with stable relations between environments (small change in place field distance). (*D*) Difference in place field distance is similar for nearby and far-away place cells. Similar to Fig. 4 *B* and *D*, a plot showing the median difference in place field distance (red) from environments *A* to *B* between a reference place cell and all its neighboring place cells as a function of incremental anatomical distance to the neighboring place cells, together with the 99% CI at each distance (shaded red area) and the median of a randomly picked control (blue).

Previous studies with smaller cell samples have reported stability of functional clusters across environments in delayed-matching to sample and foraging tasks (26). Therefore, in the present study, we looked for a relationship between anatomical position and place-field position across recording environments by comparing the relation between pairs of place cells in two environments, referred to as A and B. First, as expected, we observed clear global remapping between place cells in A and B but not across repeated tests within A (A-A’) (Fig. 6*A*). Whereas many cells displayed a change in preferred firing position in A and B (Fig. 1 *F*, *Top* row), others were silent in one environment and spatially tuned in the other (Fig. 1 *E*, *Middle* and *Bottom* row). For all place cells with stable tuning in both environments (n = 4,140, *Materials and Methods*), we compared the correlation of tuning maps between environments and their respective randomly chosen controls (n = 100 iterations). As a complementary measure of remapping, we performed the same comparison by comparing the distance by which the center of mass for each tuning map shifted between environments. Only correlations upon re-exposure of the same environment (A-A’) stood out as different from random control samples [Fig. 6*A*; median correlation of A-A’ with 99% CI from bootstrapping with 10,000 resamples: 0.75 (0.70, 0.79)]. The correlations between spatial tuning maps in A and B (median = −0.11) did not differ substantially from the correlation of A with the random controls of A (median = −0.080) or B (median = 0.081). Concordantly, the center of mass shifts for the spatial tuning maps were low for place cells from A to A’ [Fig. 6*A*; median center of mass shift of A-A’ with 99% CI from bootstrapping with 10,000 resamples: 7.50 (6.20, 8.83)]. The shifts in center of mass were substantially higher from A to B (median = 30.4 cm) and were comparable to that of A versus the randomly chosen distributions of A (median = 27.8 cm) or B (median = 26.6 cm). The remapping was not due to mere rotation of spatial tuning maps (*SI Appendix*, Fig. S1*C*). Taken together, these analyses indicate that spatial tuning map correlations and center of mass shifts between A and B are no more similar than in a comparison with a random distribution.

If place cells are organized topographically by their place fields, this organization should be maintained across different environments and thus be stable after the remapping. To test whether the relation between these stable place cells persisted after remapping, we looked at pairwise relations between the place cells. First, we calculated the pairwise distance between place fields for all pairs having a firing field within both environment A and B. Second, we calculated the difference between the place field distances for each pair between A-B, and A-A’. For these pairwise relations, the difference in place field distances remained constant from A to A’ but not from A to B (Fig. 6*B*; Levene’s test of differences in variances between A-A’, A-B, and A-Random A’: F = 30,962, df = 2, *P* < 10^−6^). The variance of place field distance from A to B was comparable to that from A to a randomly chosen control cell of A’. Furthermore, when we compared the pairwise place field distances from A-B to the anatomical distance between each pair, there was no relation between anatomical distances and difference in place field distances (Fig. 6*C*; mutual information for continuous variables: MI = 0.00076, n = 176,424 pairs). Also, there is no subpopulation of neighboring place cells (small anatomical distance) with stable relations between environments (small change in place field distance). Together, these pairwise approaches did not uncover a maintained relation of functional tuning among place cells after remapping.

Finally, to look for relations within small ensembles of neighboring place cells, we followed a similar approach as in Fig. 4 *B* and *D*. As above, only place cells active in both environments were considered. For each of these place cells, we calculated the differences in place field distances between a reference place cell to that of all its nearby neighbors while gradually increasing the distance from the reference cell determining the local ensemble. We then compared the median difference in place field distances across distances from the reference place cells and calculated the 99% CI (from 10,000 bootstraps) at each distance. This was compared to size-matched randomly chosen controls of cells at all distances. The random control sample fell within the CI at all distances (Fig. 6*D*). Also, the differences were similar for nearby and far-away cells (Cliff’s delta for effect size between place field distance at 15 µm and 60 µm: d = −0.044, n = 13,416). Taken together, and in agreement with studies showing a fully orthogonal relation between place-cell maps for different environments (43–45), these analyses gave no indication that small clusters of place cells retain spatial relations after global remapping.

### Anatomical Distance Has No Impact On Accuracy of Position Decoding.

Next, we asked whether population analyses of place cells might reveal an underlying topographic organization not seen in pairwise comparisons. If CA1 place cells were clustered, with high functional tuning similarity among nearby neurons, the activity in a cluster of cells would not inform as accurately about the animal’s position as a fully distributed cell sample. To test this prediction, we decoded the animal’s position based on activity from subsets of nearby place cells and compared it to the decoding error obtained when using activity from a size-matched subset of place cells randomly selected from the FOV within the same session. We built a Bayesian decoder to estimate the animal’s position by training it on the place cells’ activity and the animal’s position in every other temporal bin. When testing its performance on the remaining bins, the decoder could accurately determine the actual position of the animal, far above chance levels (*SI Appendix*, Fig. S7). Furthermore, as expected, when decoding from subsets of only similarly tuned place cells, the decoding error increased compared to when we decoded on equal numbers of randomly selected place cells (*SI Appendix*, Fig. S8).

**Fig. 7.**
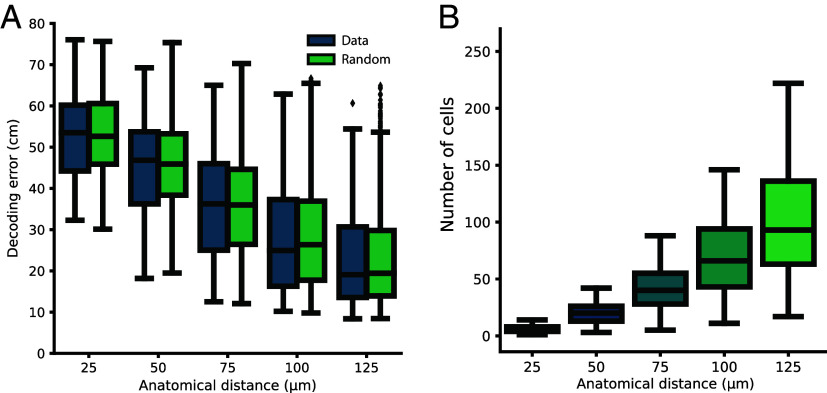
Positional decoding from CA1 place cells. (*A*) Position decoding from neighboring place cells is not impaired compared to decoding from randomly selected place cells. Boxplots show decoding error as a function of anatomical distance for place cells within given anatomical distance bins (“Data,” blue) and for a size-matched randomly picked control (“Random,” green) at each distance. As the anatomical distance increases, the number of cells used to decode position increases and the decoding error gradually lowers, as expected. However, there is no noticeable difference between the data and the randomly picked control group. Symbols as in Fig. 3*D*. (*B*) Boxplot showing number of cells as a function of anatomical distance for randomly selected place cells.

Using the decoder, we started with a single reference place cell and defined its local neighborhood by an incremental anatomical distance. Place cells that fell within this distance were included in the decoder. The procedure was performed for 200 reference place cells for 24 sessions (environments A and B in 12 sessions), each tested for five anatomical distances. Each test was compared to 25 size-matched subsets of randomly picked place cells from the same session. When the anatomical distance increased, and thereby also the number of cells included, the decoding error decreased, as expected, for both the underlying data and the random control sample (Fig. 7 *A* and *B*). However, there was no difference in decoding error between anatomically nearby cells, within any radius, and randomly selected place cells (median decoding error of nearby place cells with 99% CI included the median decoding error from randomly selected cells at all anatomical distances, see *SI Appendix*, Table S1). Taken together, the decoding approach showed no difference in accuracy related to the relative anatomical location of the place cells.

### Object-Tuned Cells in CA1 Are Also Not Topographically Organized.

**Fig. 8.**
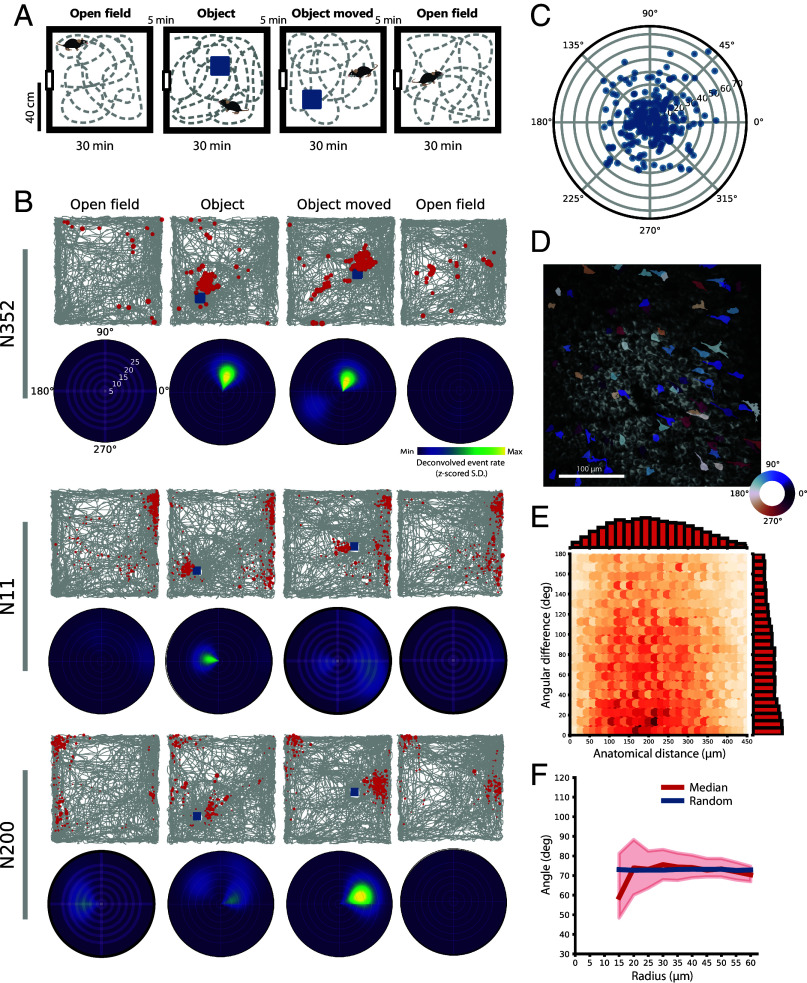
Object-tuned cells in CA1 are not topographically organized. (*A*) Experimental setup for measuring object tuning in CA1. Mice explored the same open field four times. During the second session, a high-contrast and nonclimbable object (depicted as a blue square) was introduced into the arena. The object was moved before the third session and removed before the final session. (*B*) Trajectory maps (square) and object-centered tuning maps (circular) for cells in CA1. For the trajectory maps, the trajectory of the mouse is plotted in gray for each of the four sessions (columns). Deconvolved calcium events are superimposed in red. The object is marked as a blue square. Object-centered tuning maps show activity with reference to the object, with the object at the center of the plot, showing tuning to angle (deg) and distance (cm) from the object. The activity (A.U.) is normalized per cell (Scale bar as in Fig. 3*C*). Cell “N352” has low activity in the empty open field, but a strong field emerges in the object sessions. The new field’s relation to the object is maintained from “Object” to “Object moved.” Cell “N11” has a place field in one corner that is apparent in both open fields, but the cell also gains an object field with a maintained relation to the object in “Object” and “Object moved.” “N200” has a place field in the *Top Left* corner of the box and gains a stable object field in the object sessions. (*C*) Polar scatter plot showing object tuning for all object-tuned cells. Preferred angle and distance from the object are shown for each cell (one dot per cell, data across four experiments). (*D*) Example FOV from a mouse showing the distribution of object-tuned cells within the FOV. ROIs are color coded by their preferred angular tuning (color scale at the *Bottom Right* corner). Object-tuned cells appear scattered around the entire FOV, and cells with different angular tuning appear intermingled. (*E*) Heatmap showing angular tuning as a function of anatomical distance for all pairs of object-tuned cells. Heatmap like Fig. 2 *B* and *C*; data pooled over four sessions (n = 14,504 pairs). There is no clustering of anatomically close object-tuned cells with low angular difference. (*F*) Angular tuning for object-tuned cells is similar for nearby and far-away cells. As in Fig. 4 *B* and *D*, the plot shows the median of preferred angular tuning (red) of nearby object-tuned cells with its 99% CI (red shaded area) as a function of incremental anatomical distances from a reference cell, together with the median of a randomly picked control (blue).

To further test if there is clustering of cells with similar functional properties in CA1, we turned our attention to cells with specific tuning to salient local objects or “landmarks” (46–49), whose organization might potentially mirror the reported anatomical clustering among reward-tuned cells (29). A total of three mice underwent experiments in which the animal ran in an open field environment for four sequential sessions, during which a high-contrast nonclimbable object was placed at two successive locations on the second and third session (“Object” and “Object moved”, Fig. 8*A*). Object-tuned cells, including “landmark-vector cells” (46), were classified as cells that developed a new and stable activity field at similar distance and direction from the object on the two sessions with objects (46–48) (cells with firing locations at the object were included; for details, see *Materials and Methods*). By this approach, we identified 328 object-tuned cells among a total of 3,254 recoded CA1 cells (10.1%), with a range from 48 to 120 object-tuned cells per experiment (8.0 to 13.2% of cells) in a total of four experiments (one animal was recorded twice with different FOVs). The fraction of object-tuned cells is comparable to observations in CA1 of rats (49). In the present study, a total of 212 object-tuned cells (65%) satisfied criteria for place cells in the initial trial with no object. On the Object trial, the object-tuned cells acquired a new field with coordinates determined by the object (Fig. 8*B*, middle row). In the majority of object-tuned cells, the firing field was near the object (median distance from center of object: 14.9 cm, IQR: 8.6 to 25.7 cm), although a few were located further away (Fig. 8 *B*, *Bottom* row), mirroring object-vector cells in MEC (47). The population of object-tuned cells covered all directions from the object uniformly (Rayleigh test of circular uniformity: Z = 1.03, *P* = 0.36), and 80% of the cells had an object field within 15 cm from the object (Fig. 8*C*).

Having described a population of object-tuned cells in CA1, we next showed that these cells were scattered within the FOV with no obvious anatomical clustering of cells with similar angular tuning to the object (Fig. 8*D*). There was no relation between the angular difference of preferred tuning between pairs of object-tuned cells and their anatomical distance in the FOV (Fig. 8*E*; mutual information for continuous variables: MI = 0, n = 2,472). There was similarly no relation between the anatomical distance of cell pairs and their object fields’ distance from the object (*SI Appendix*, Fig. S9). Last, we analyzed the preferred angular tuning of local clusters of object-tuned cells by incremental anatomical distances from a single reference cell (Fig. 8*F*, similar to the approach in Fig. 4 *B* and *D*). Nearby object-tuned cells were no more similar in their angular tuning than far-away cells (for all anatomical bins, the median of randomly picked controls fell within the 99% CI of the bootstrapped median; Cliff’s delta for effect size between preferred angular tuning at 15 µm and 60 µm: d = −0.034, n = 2,472). Taken together, these analyses show that also object-tuned cells in CA1 lack a topographic organization.

## Discussion

Using multiple methods for estimating spatial topography in large samples of in total 6,519 CA1 place cells, we found that for neither place cells nor object-tuned cells were there any indications of anatomical clustering. There was also no tendency for the relation of spatial properties of anatomically adjacent place cells to be transferred between environments during remapping.

Previous studies have reported that place cells are organized in local clusters with spatial tuning properties more similar than those of more distant cell pairs (25–30). In the present study, we searched specifically for similarities in nearest-neighbor groups of place cells. When dividing the FOV into square bins, place cells within one bin were no more correlated in spatial tuning properties or calcium signals than a distribution of randomly selected cells. When a place cell’s spatial tuning was compared to that of cells in its local neighborhood by gradually increasing distances from the cell in circular bins, the spatial tuning was no more similar for nearest neighbors than for distant neighbors and it was indistinguishable from a randomly picked distribution at all distances. A similar lack of clustering was found when cells were compared to their nearest neighbors irrespective of distance, suggesting that the negative findings above were not caused by choice of too large bins or bins that cut across clusters. Consistent with these multiple observations, we could decode position just as precisely from anatomically nearby place cells as for randomly selected place cells, indicating that the firing fields of small anatomical clusters of place cells cover the environment as effectively as randomly chosen place cells. Taken together, our data provide no indication of position-dependent clustering among CA1 place cells. A similar lack of topographical organization was observed in the subset of object-tuned neurons, including landmark-vector cells, in the CA1.

These results add insight to a mix of studies of functional clustering in the hippocampus, some reporting correlations between neighboring CA1 neurons (25–29) and some not (24, 50). The inconsistencies may reflect differences in statistical power, spatial scale, and spatial resolution of activity measures. In the earliest studies, cell samples were small and data were collected with electrophysiological methods lacking single-cell spatial resolution. More recent studies used calcium imaging to map activity across larger numbers of cells with more precisely described anatomical location. Yet these studies also suffered from the limitations of the methods at the time they were conducted, such as lower numbers of simultaneously recorded cells and lower anatomical precision. The present study took advantage of more recent methodology to readdress the question of local topography in place cells of unrestrained mice. First, we used a more sensitive fluorescent indicator GCaMP6 than some of the earlier work, and transgenic mice instead of viral vectors to ensure that the indicator was expressed throughout the excitatory cell population (more than 80% of NeuN+ cells). Second, we utilized a miniature 2P microscope (MINI2P) to image, in freely moving mice, the activity of large neural populations at high spatial resolution, sufficient to distinguish neighboring neurons within a densely packed and labeled neuron population. MINI2P enabled 2P imaging without the constraints of head-fixation and its possible impacts on position computation (32, 33). Third, the low photobleaching of 2P compared to 1P allowed us to record over several hours, long enough to compare activity across environments and behavioral tasks. Finally, to achieve high-resolution, large-scale neural population recordings in the hippocampus, we refined a custom-designed glass plug to serve as an imaging window for the underlying CA1 neurons. The glass plug presents an improvement over GRIN lenses, which are commonly employed for 1P miniscope imaging (36), by offering an expanded FOV with reduced optical aberrations. When compared to the large-diameter cannulas used in prior head-fixed 2P studies (30), the glass plug is notably smaller in diameter, reducing the required aspiration volume and thereby minimizing surrounding brain damage.

Together, these methodological amendments allowed us to investigate the functional microstructure of CA1 place cell networks during unrestricted foraging. Using several analytical approaches, we found consistently that the firing locations of neighboring CA1 place cells were no more similar than those of randomly selected place cells. This does not exclude the possibility of topographical encoding of higher-level variables, such as reward-encoding, for instance through sparse dopaminergic inputs to CA1 (29, 51), nor does it rule out the possibility that clustering appears under certain behavioral conditions such as when animals run on linear tracks (29, 30), although it would not be clear what the mechanism for such dynamic clustering could be. The most parsimonious interpretation of the present findings is that the hippocampal spatial map is fully nontopographical.

Finally, since representations of space in CA1 place cells are usually orthogonal between different environments (43–45), a fixed anatomical relationship between neighboring place cells would have reduced CA1’s capacity for storing very large numbers of independent representations as the combinatorial power of place-cell activations would be restricted. Although quantitative studies would be required to determine the extent of local clustering needed to disrupt storage capacity in sparsely coding networks, our finding of a fully nontopographic place cell spatial map in which the spatial correlation structure of neighboring place cells is not retained across environments, is consistent with the supposed ability of the hippocampus to store vast numbers of unique neural representations (52). In such terms, our demonstration of a lack of functional clustering supports the hippocampus’ role in high-capacity episodic memory formation (53–55).

## Materials and Methods

### Mice and Surgery.

All experiments were performed using transgenic Thy1-GC6 mice (C57BL/6 J-Tg(Thy1-GCaMP6s)GP4.3Dkim/J) (34, 38). A custom glass plug assembly (glass plug glued to a glass coverslip) was implanted in the right hemisphere of eight adult mice following procedures previously described (35, 37). The cortex above the exposed dorsal CA1 was carefully aspirated, but the alveus was kept intact (36). After the aspiration, the glass plug with coverslip was slowly inserted into the aspirated area with a stereotactic frame until the glass plug made full contact with the exposed CA1. The whole assembly and a metal head bar were attached to the skull with adhesives and dental cement (37). A miniature two-photon microscope (miniscope) was mounted 2 to 3 wk after surgery. Then, the animals underwent pretraining by handling, familiarization, and running in an open field with a dummy scope.

### Spatial Foraging Task.

Mice were trained to run in an open field with a square box of 80 cm × 80 cm with 50 cm high walls. A cue card was used for orientation. The two environments were located within the same room, and differed in color and external cues. Both environments were enclosed with curtains. The animals usually ran for 35 min in each session.

### In Vivo Two-Photon Imaging With Miniscopes.

Two-photon imaging was performed by a miniaturized two-photon microscope (MINI2P) using a fiber-based femtosecond pulsed laser at 920 nm wavelength (Ultra-920, Toptica, Munich, Germany) and a 0.45 NA objective optimized for imaging through 1.5 to 2.0 mm thick glass (35). Output power, measured after the miniscope objective, was usually around 60 mW. The animal’s behavior during the tasks was tracked by an infrared camera mounted on the top of the experiment box which was synchronized with the two-photon imaging. Body parts (ears, head center, body center, tail root) of the animals in each frame of the tracking movie were detected using DeepLabCut (56). Position and head direction of the animal were calculated using DeepLabCut, using methods previously reported (35). Scanning distortion was corrected for all data such that the spatial scale across the entire imaging FOV was constant, and linearly proportional to real anatomical distances. Motion correction, ROI extraction, calculation of signal traces and deconvolution were performed by the Python based version of suite2p (57). Calcium signal and deconvolution were calculated through suite2p as described in *SI Appendix*, *Materials and Methods*. Each cell’s signal-to-noise ratio (SNR) was calculated by defining “signal” as the mean amplitude over all 90th percentiles of ΔF/F(t) during significant transients and “noise” as the noise level of ΔF/F(t) calculated as the mean of differences of ΔF/F(t) for instances outside the significant transients. Only cells exceeding an SNR of 3 were used in further analyses.

### Spatial Tuning Maps and Allocentric Object Tuning Maps.

To analyze spatial activity properties of neurons, spatial tuning maps were calculated. The environment was divided into 2.5 cm × 2.5 cm spatial bins, and the deconvolved calcium events that occurred within each spatial bin were summed and divided by time spent in that bin. The activity rate map was smoothened by a Gaussian 2D kernel (σ = 2.5) and speed-filtered.

For analyses of tuning to objects, allocentric object-tuning maps were calculated. These describe cell activity as a function of the animal’s distance away from (in 2 cm bins) and angle to (in 6° bins) the object at each timepoint. For these maps, the sum of calcium events within each bin was divided by the time spent in each bin to get an activity rate. This activity rate was smoothened similarly to the spatial tuning maps.

### Place Cell Classification.

Place cells were defined based on multiple criteria (35, 58). To be considered for analyses, the cell needed to have an SNR > 3 and at least 40 calcium events within an environment. Cells meeting these criteria were classified as place cells based on spatial information, stability within the session, and existence of one or several discrete place fields.

### Analyses of Spatial Clustering.

To analyze if the spatial tuning of local clusters of place cells was similar, we compared the spatial tuning of a “reference cell” to that of all neighboring place cells within an incremental anatomical distance from the reference cell’s anatomical location. Further, clustering by correlations of place cell maps across anatomical bins was determined using Global Moran’s I, a measure of spatial autocorrelation that quantifies how scattered or clustered a variable of interest is in a two-dimensional area (42). Significance was calculated by randomly reassigning the correlation value for each bin and recalculating the Moran’s I for 1,000 iterations.

## Supplementary Material

Appendix 01 (PDF)

## Data Availability

All code used for data analysis is available at GitHub (https://github.com/torsteinsl/ca1topography) (59). The data are made available via DOI (https://doi.org/10.25493/TRTW-NK8) (60).
