# Supplementary material for: Place cells in CA1 lack topographical organization of firing locations
Source: Proc Natl Acad Sci U S A. 2026 Feb 18;123(8):e2528601123. doi: 10.1073/pnas.2528601123 (PMC12933062; doi:10.1073/pnas.2528601123)
Supplement: Supplementary file 1 — Appendix 01 (PDF) [file pnas.2528601123.sapp.pdf]

Place cells in CA1 lack topographical organisation of firing location

Torstein Slettmoen[1], Nienke L. de Jong[1], Hanna Eneqvist[1], Emilie R. Skytøen[1], Weijian Zong[1], May-Britt Moser[1], Edvard I. Moser[1]*

[1] Kavli Institute for Systems Neuroscience and Centre for Algorithms in the Cortex, Norwegian University of Science and Technology, 7491 Trondheim, Norway.

** Corresponding author: E.I.M., address as above.*

This PDF contains:

Materials and methods

Supplementary Figures 1-9

Supplementary Tables 1-2

Materials and methods

Ethical considerations

All experiments were performed in accordance with the Norwegian Animal Welfare Act and the European Convention for the Protection of Vertebrate Animals Used for Experimental and Other Scientific Purposes. All experimenters were FELASA-C certified. The 3Rs were applied to all aspects of animal housing, surgery and experiments. The experiments were approved by the Norwegian Food and Safety Authorities and reported under FOTS number 18013 and 29894.

Mice

Mice were kept in enriched cages, containing running wheels, cotton, chewing sticks and different houses or shelters, with availability of food and water ad libitum at a reversed 12-hour day-night cycle and with controlled temperature and humidity. Housings were free of specific pathogens in accordance with the Federation of European Laboratory Animal Science Associations. From implantation mice were housed separately. All experiments were performed using transgenic Thy1-GC6 mice (C57BL/6J-Tg(Thy1-GCaMP6s)GP4.3Dkim/J) from the Jackson Laboratory, JAX stock #024275) (*1, 2*). This mouse line has high expression of GCaMP6s in the hippocampus, and our histological data are consistent with the reported expression from the mouse line (*2*), in which the expression across CA1 is abundant and covers most excitatory neurons (79-86 % across individuals) along both the transverse and longitudinal axes of the CA1 (see Supplementary Fig. 2). In total, eight mice were used in this paper (four females and four males), seven of which underwent experiments and are presented here, whilst one was implanted but did not grant any data.

Surgery: Aspiration and CA1 implantation

Adult mice had to be at least 3 months of age to undergo surgery. Anaesthesia was induced with 5 % isoflurane in an induction chamber at flow 2 L/min. When anaesthetised, mice were given 0.5 mL of hyperosmotic mannitol (Mannitol) 150 mg/mL ip. Analgesics were delivered sc. as buprenorphine (Temgesic) and meloxicam (Metacam) together with local anaesthetics of the scalp with bupivacain (Marcain). Animals were put in a stereotactic frame with a face mask delivering isoflurane at 1-3 % at approximate 1L/min flow based on respiration depth and frequency, together with oxygen at 0.6-1 L/min flow from an oxygen concentrator. The scalp was incised, and the animal head-fixed and aligned with lambda and bregma. A craniectomy was performed using a trepanation drill of diameter 1.8 mm, centred at coordinates: AP: -2.10 mm from bregma; ML: 1.60 mm laterally from the midline; angle: 10° tilt medially. All mice were implanted in the right hemisphere. The cortex above the dorsal CA1 was carefully aspirated (*3*), matching the craniectomy in size, using a BVC Professional Fluid Aspiration System. Aspiration was performed under constant irrigation of cool saline, until fibres could be detected. The most superficial fibres (running in mediolateral direction) were carefully aspirated so that the anteroposterior running fibres were visible. Thus, the alveus was kept intact. The aspirated cavity was filled with Spongostan until all bleedings stopped. Then, a premade disinfected implant with a glass plug (1.80 mm diameter, 1.50 mm hight), comparable to those previously reported (*3, 4*), glued to a glass cover slip and a flat head bar was lowered into the aspirated area with a stereotactic frame. The implant was secured to the skull, first using OptiBond and Charisma Diamond together with Venus Diamond Flow, and then dental cement (Meliodent) mixed with carbon powder to make the cement dark and less reflective. Lastly, the cover slip was protected from the outside with Kwik-Cast to protect direct light exposure and dust accumulating over the area to be imaged.

Postoperatively, animals recovered in a heating chamber until awake and alert. Analgesics were administered 8 hours postoperative as buprenorphine, and 24 hours postoperative as meloxicam.

After surgery, mice were kept in single cages and were given 2-3 weeks of rest before experiments started.

Pretraining

After 2-3 weeks from surgery, animals were tested head-fixed on a running wheel under a miniaturised two-photon microscope to check for GCaMP-signal. Animals in which GCaMP was expressing in several cells (seven out of eight animals) were baseplated and underwent pretraining by handling, familiarisation and running in an open field without a tether, and having a dummy scope attached in the home cage. Training was performed in box A.

Spatial foraging task

Mice were trained to run in an open field with a square box of 80 cm x 80 cm with 50 cm high walls chasing cookie crumbles. For the remapping experiments, box A was all black with a white cue card, whilst box B was all white with a black cue card. The two environments were located in different parts of the recording room, approximately 2 meters apart in a 5 m x 8 m room, and had similar orientation of local cues, yet different distal cues. Both environments were enclosed with curtains. Experiments were conducted successively without removing the miniscope from the animal. The animals usually ran for 35 min in each of the sessions A, B and A', with a 30 min break in their home cage in between sessions in which they had access to water and food.

For the object experiments, mice ran in a similarly shaped box (80 cm x 80 cm) over four sessions: open field, object, object moved and open field. Each session lasted for about 30 minutes, with an 5-10 minute pause in between where the animal was in its home cage, with the miniscope still attached. The object was a high contrast yellow tower of dimensions 7 cm x 7 cm with height 27 cm. The object was both introduced to the arena and moved at least 20 cm while the animal was in its home cage in between sessions, yet not put too close to walls to not lose possible activity fields that would end up outside the box.

In vivo two-photon imaging with miniscopes

Two-photon imaging was performed by a miniaturised two-photon microscope (MINI2P) using a fiber-based femtosecond pulsed laser at 920 nm wavelength (Toptica, Ultra-920) and a 0.45 NA objective optimised for imaging through 1.5-2.0 mm thick glass (*5*). MINI2P allowed high resolution imaging in freely moving mice with a post distortion correction FOV of ca. 350 x 350 µm, imaged at resolution 512x512 pixels at 7.5 Hz. For choosing the FOV to record, we first head-fixed the animal on a running wheel, started MINI2P imaging, and moved the miniscope with a three-axis motorised stage above the imaging window (the glass plug implanted above CA1) while evaluating the imaging sharpness, cell number, and spontaneous activity level in each position. As soon as the position was deemed satisfactory, the baseplate onto which the miniscope was to be mounted was cemented onto the headbar. During the imaging experiments, laser power and PMT (ThorLabs, PMT2101) gain were adjusted to the acquired image to give satisfactory signal without introducing significant bleaching across the entire recordings. Output power, measured after the miniscope objective, was usually around 60 mW. Imaging acquisition was performed using the ScanImage Software (Version 2022, MBF). The animal´s behaviour during the tasks was tracked by an infrared camera mounted on the top of the experiment box (Basler, acA2040-90umNIR). The animal tracking camera was synchronized with the two-photon imaging such that each two-photon imaging frame triggered one tracking camera acquisition (7.5Hz). The position of the animal in the open field was extracted from the tracking movie through deep-learning-based pose-extraction algorithms in DeepLabCut (*6*).

Distortion correction

Distortion correction was performed as previously reported (*5*). Since the MINI2P generates a distorted image towards the borders of the FOV, a distortion matrix was created by imaging a fluorescent sample carrying a square grid pattern using MINI2P. As the level of distortion changes with the level of voltage through the tuneable lens (µTlens, for details see (*5*)), the distortion matrix was created for 24 *z*-levels spanning the 0-240 µm *z*-range. From our imaged grid sample, anchor points were generated at each crossing of vertical and horizontal lines. These points were manually adjusted to each level of the tuneable lens. From these adjusted points a linear transformation matrix was generated in MATLAB. This matrix was then applied to the raw image data to correct for distortions. All data included in this paper have been distortion corrected such that recorded anatomical distances are true to real anatomical distances.

Motion correction and ROI extraction

Motion correction, ROI extraction, calculation of signal traces and deconvolution were performed by the Python based version of suite2p (https://github.com/MouseLand/suite2p) (*7*). Distortion corrected images were analysed with suite2p (of which key parameters were: τ, timescale in seconds for the deconvolution kernel = 1.5; smooth_sigma, standard deviation of the gaussian kernel used for pixel smoothing for the motion correction = 1.15). Motion correction was performed non-rigidly (*7*). ROI extraction was based on the functional channel. The putative ROIs were then manually curated by the experimenter using the suite2p GUI, basing the cell selection of ROIs resembling cells (round and preferably with a nuclear clearing) and the calcium trace resembling cell activity (fast high peak, slow decay). The curated ROIs were then inserted further into an analysis pipeline.

Calcium signal and deconvolution of neural activity

Calcium signal and deconvolution was calculated through suite2p. This yields three outputs of calcium traces per ROI detected: the raw fluorescent trace of the cell ($F_c(t)$), the neuropil ($F_{np}(t)$), and the deconvolved calcium activity ($D_c(t)$). The corrected fluorescent trace per cell was calculated by subtracting a constant coefficient of the neuropil from the raw calcium signal ($F_{corr} = F_c(t) - 0.7\ F_{np}(t)$). Deconvolution was computed by non-negative deconvolution of the F_{corr}, using a decay constant exponential kernel matching our calcium indicator (τ = 1.5 seconds) (*8*). Deconvoluted signals were used to increase temporal precision and denoise the fluorescent signal.

To further tackle fluctuations within the fluorescent signal (F_{corr}), the suite2p output was further analysed using the openly available MATLAB application NATEX (*5*). These calculations consider both the effect on the calcium signal from potential photobleaching over time and possible noise causing fluctuations rather than actual neuronal activity:

First, to lower potential effects of slow variation of the baseline signal caused by drifting or photobleaching, a running baseline of F_{corr}, denoted $F_0(t)$, was calculated. As described by others, the baseline $F_0(t)$ is given by $F_s(t) + m$, where $F_s(t)$ is the eighth percentile of the $F_{corr}(t)$ within a ± 15 seconds time window centred at t, and m is a constant added to $F_s(t)$ so that $F_0(t)$ is centred at 0 when there is no calcium activity. The baseline time points were defined at instances where the local standard deviation (std) of the signal (± 15 seconds time window of $F_{corr}(t)$) did not exceed a constant of the global standard deviation (all datapoints) given by: $std_{min} + 0.1\ (std_{max} - std_{min})$. Then, the change in fluorescent signal was computed as follows:

$$\frac{\Delta F}{F(t)} = \frac{F_{corr}(t) - F_0(t)}{F_0(t)}$$

Secondly, to filter out instances in which changes in the calcium signal corresponds to actual activity of the neuron rather than artificial fluctuations, for instance due to electrical noise or motion artefacts, significant transients were computed. Significant transients were defined as transients that exceed the local std of $F_0(t)$ by at least a factor of 2 for at least 0.75 seconds. Instances in which the $\Delta F/F(t)$ fulfilled this criterion were considered as "significant", and both the $\Delta F/F(t)$ and the $D_c(t)$ were then filtered by these instances. Lastly, both these filtered signals were normalised to the maximum of the filtered $\Delta F/F(t)$. Non-zero instances of the filtered deconvolved signal were referred to as "calcium events", and all spatial tuning maps in this paper were calculated using this signal.

Each cell's signal-to-noise ratio (SNR) was calculated, in which "signal" was defined as the mean amplitude over all 90th percentiles of $\Delta F/F(t)$ during significant transients and "noise" was the noise level of $\Delta F/F(t)$ calculated as the mean of differences of $\Delta F/F(t)$ for instances outside the significant transients. Only cells exceeding an SNR of 3 were used in further analyses.

Perfusion and histology

Mice were euthanised under general anaesthesia with isoflurane by an intraperitoneal injection of overdosed pentobarbital (Pentobarbital). Death was confirmed with respiratory stop and loss of brainstem reflexes. Perfusion was performed first by transcardial cannulation, lacerations of the liver and flushing with isotonic NaCl. When blood was flushed out, the animal was perfused with a fixative of freshly made 4 % paraformaldehyde (PFA) in phosphate-buffered saline (PBS). After perfusion the brain was extracted and kept in PFA for 24-48 hours. After fixation, brains were cut sagittal on a cryostat (Thermo Scientific Micom HM 560) as frozen sections of 50 µm. Only the implanted hemisphere was cut. Sections were kept in phosphate buffered saline (PBS) for immunostainings.

Immunostaining

Sections were immunostained within days after sectioning. Immunostainings were performed to identify GCaMP6 (GFP) and visualise neurons (NeuN) in the area recorded. First, sections were preincubated in a PBS solution with 0.3 % Trition-Tx and 3 % bovine serum albumin (BSA) for 1 hour at room temperature. Secondly, sections were incubated with anti-GFP chicken (AbCam, 13970, LOT: GR3361051-7, 1:1000) and anti-NeuN rabbit (AbCam, ab177487, LOT: GR3275122-15 and GR3393363-5, 1:1000) as primary antibodies for 12-18 hours at room temperature. Then, sections were washed with PBS with 0.1 % Triton-Tx and 1 % BSA. Third, sections were incubated in PBS with 0.1 % Triton-Tx and 1 % BSA with anti-chicken 488 (Alexa Fluor 488 goat anti-chicken, ThermoFisher, A-11039, 1:750) and anti-rabbit 647 (Alexa Fluor 647 donkey anti-rabbit, ThermoFisher, A-31573, 1:750), as secondary antibodies for 1.5 hours at room temperature. Sections were washed with PBS, then mounted on slides and covered with ProLong Gold AntiFade. Epifluorecent images were acquired with an Axio Scan Z1 microscope with Zen software (Carl Zeiss) with 10x magnification. Confocal images, used for cell counting, were acquired using a Zeiss LSM 880 microscope (Carl Zeiss, Germany) with ZEN pro software, with 40x magnification. The acquisition was adapted to the fluorophores, with similar laser power and PMT gain across sections for each animal.

Spatial tuning maps and allocentric object tuning maps

To analyse spatial activity properties of neurons, spatial tuning maps were calculated. The environment was divided into 2.5 cm × 2.5 cm spatial bins, and the deconvolved calcium events that occurred within each spatial bin were summed. The value for each bin was divided by time spent in that bin to compute the activity rate within the bins. This activity rate map was smoothened by a Gaussian 2D kernel ($\sigma = 2.5$). Only bins which the animal visited for at least 0.1 second were analysed,

and non-visited bins were kept out of the analyses. To analyse times in which the animal was foraging, only instances in which the moving speed of the animal was at least 2.5 cm/s were analysed.

For analyses on object tuning, allocentric object tuning maps were calculated. These are centred around the object and quantify the cells activity as a function of the animal's distance away from (in 2 cm bins) and angle to (in 6° bins) the object at each timepoint. The sum of calcium events within each bin was calculated and divided by the time spent in each bin to get an activity rate. The activity rate was smoothened by a Gaussian 2D kernel ($\sigma = 2.5$). Only instance in which the mouse was moving at least 2.5 cm/s were analysed.

Place cell classification

Place cells were defined based on multiple criteria, based on a combination of previously reported methods to define place cells and on the method published for similar MINI2P data (*5, 9*). To be considered for analyses, the cell needed to have an SNR > 3 and at least 40 calcium events within an environment. Cells meeting these criteria were tested within each environment for the following: Spatial information, stability, and existence of a place field.

1) Spatial information (SI): The spatial information of a cell was calculated based on previous reported methods (*10*). SI was calculated in bits per $\Delta F/F\ s^{-1}$, as:

$$\sum_{i=1}^{n} p_i \frac{\lambda_i}{\lambda} \, log_2 \frac{\lambda_i}{\lambda}$$

   where λ_i is the mean amplitude of calcium activity in the *i*-th bin, λ is the overall mean amplitude of calcium activity, and p_i is the probability of the animal being in the *i*-th bin. For each cell, the cell's SI was compared to a shuffled distribution in which the cell's calcium events were randomly circularly shuffled in time for 500 iterations, and for each iteration the shuffled data's spatial tuning map and SI was calculated. Calculations were performed using the Opexebo Python package (https://github.com/simon-ball/opexebo/blob/master/docs/index.rst). Only cells in which the SI exceeded the 95th percentile of the SIs of the shuffled distribution were considered significantly spatially tuned to be considered a place cell.
2) Stability within a session: To test whether the cell's spatial tuning was stable within one environment, as expected by place cells, the session was split in halves. Spatial tuning maps for each half were calculated, and the first and second halves' spatial tuning maps were correlated. This was compared to the correlation between the first and second halves of the recording when shuffling the calcium events for 500 iterations as in (1), and only cells exceeding the 95th percentile of the shuffle distribution were considered as place cells.
3) Place field detection: The spatial tuning maps for each cell were used to identify place fields. Only cells with detectable place fields were considered as possible place cells. The field detection analysis was performed using the Opexebo Python package. Fields were identified using an adaptive threshold, gradually decreasing from a peak value. The field would increase in size as the bin-wise activity was still above the adaptive threshold. The field was fully defined when lowering this threshold did not expand the field. Fields with peak activity lower than 0.2 deconvolved calcium events per spatial bin were ignored. Cells in which no field was detected in the tuning map were discarded. For place cells with multiple detected fields, the cells' main field was defined as the field with highest mean deconvolved activity (A.U). For analyses of place fields, the main field was used unless stated otherwise, with the location of the field determined by its centroid.

Only cells exceeding the signal quality criteria (SNR and number of calcium events) and the three above criteria (spatial information, stability, and place field detection) were defined as place cells.

To analyse if relations in spatial tuning after remapping were maintained between place cells, only cells that were classified as place cells in all three environments were considered. Then, to test if the cells showed stable firing between environment A and A', the correlation between the two spatial tuning maps was compared to that of a shuffled distribution as described under point 2 above. Here, the calcium signal from data in environment A' was shuffled 100 times, and new spatial tuning maps were calculated. The original tuning map from A was then correlated to the shuffled tuning map in A'. A place cell was defined as stable if the correlation in A to A' for the actual spatial tuning maps exceeded the 95th percentile of the shuffled distribution.

Global Moran's I

Global Moran's I is a measure of spatial autocorrelation, quantifying how scattered or clustered a variable of interest is in a two-dimensional area (*11*). The method is commonly applied in geographic studies but has also been introduced in studies on topographic organisation of neuronal populations in both CA1 and MEC (*12, 13*). Based on the value of global Moran's I, we may investigate if variables of similar magnitude tend to be closer to one another or not. The value of I ranges from -1 to 1, in which -1 corresponds to full dispersion (like colours in a chequerboard), whilst 1 corresponds to full clustering (like the colours in Monaco's flag). Thus, if the value of Moran's I is sufficiently high, this indicates that neighbouring regions within the area tend to have similar values. On the other hand, if Moran's I is sufficiently low, this indicates that neighbouring regions tend to have different values. Moran's I is defined as:

$$I = \frac{n}{W} \frac{\sum_{i=1}^{n} \sum_{j=1}^{n} w_{ij} \, (x_i - \bar{x})(x_j - \bar{x})}{\sum_{i=1}^{n} (x_i - \bar{x})^2}$$

where n is the number of datapoints, indexed by i and j, x is the variable of interest, $\bar{x}$ the mean of all values of x, w_{ij} is the weight between the given pair i and j, and W is the sum of all weights where w_{ii} = 0. Weights between the bins were defined by the inverse of the Euclidean distance between the central coordinates of the bins.

Further, the expected value of Moran's I is defined as:

$$E(I) = \frac{-1}{n-1}$$

in which the E(I) is negative yet close to 0 as n increases. If the calculated Moran's I is at level close to E(I), this indicates a random distribution of values within the area.

Here, we applied global Moran's I to analyse spatial autocorrelation of correlations of place cells´ spatial tuning maps within anatomical bins (the variable of interest, x) of approximately 45 x 45 µm across the entire FOV to evaluate whether the bin-wise correlation values are clustered or spread across the FOV. As before, if an anatomical bin had a least 3 place cells, the central coordinate of this bin was assigned the mean correlation of all pairs of place cells within this bin. Significance was calculated by randomly reassigning the correlation value for each bin and recalculating the Moran's I for 1000 iterations. If the global Moran's I for one FOV exceeded that of the 95th percentile of the random distribution, it was considered significant. All Moran's I and random distributions were computed using the ESDA open-source Python library (https://github.com/pysal).

Local Moran's I

The measure of global Moran's I may be decomposed to values of spatial autocorrelation at single points within the area of study, called local Moran's I, quantifying how similar a point's value is to its neighbouring values. The global Moran's I is related to the local Moran's I so that the average of all local values equals the global value. Local Moran's I allows localisation of possible clusters of areas with similar magnitude of the variable of interest, whereas the global Moran's I quantifies whether there are clusters, randomness, or dispersion of the variable of interest. The local Moran's I is defined as:

$$I_i = \frac{n(x_i - \bar{x})}{\sum_j (x_j - \bar{x})^2} \sum_j w_{ij}(x_j - \bar{x})$$

where symbols are as for the calculation of global Moran's I. As for values of global Moran's I, local Moran's I ranges from -1 to 1, and a high positive value of I_i indicates that datapoint *i* has neighbours of similar magnitude, whilst a low negative value indicates dispersion in which datapoint *i* is different in magnitude than its neighbours.

To assess the significance of all local Moran's Is, p-values were computed for each I_i by random reassigning variables and datapoints for 999 permutations whilst keeping x_i for region *i* constant. The p-value was determined by comparing the calculated I_i to the distribution of permuted I_i. As for global Moran's I, the computations were performed using the ESDA Python library. To correct for multiple comparisons, false discoverer rate correction was performed by the statsmodels open-source Python package (https://www.statsmodels.org).

Local topographical analyses with expanding radii

To analyse if the spatial tuning of local clusters of place cells were similar, we compared the spatial tuning of a 'reference cell' to that of all neighbouring place cells within an incremental anatomical distance from the reference cell's anatomical location. The reference cell's spatial tuning was compared pairwise to all neighbours within the expanding radii (see Fig. 4A), and the local topography was considered as the mean of all pairwise comparisons at each distance. For analyses based on only one environment, the results from both environment A and B were used (A' was not used).

Bayesian decoding of position from place cell activity

To decode the animal's position in the open field box we built a Bayesian decoder, building upon an existing approach (*14*). We assumed a flat prior and maximised the likelihood across the different classes x which represent the animal's spatial location, yielding the likelihood expression:

$$\underset{x}{\operatorname{argmax}} \, [P(\overrightarrow{pv}|x)]$$

where $\overrightarrow{pv}$ denotes the population vector of the place cells for a given time bin. To calculate the likelihood, we assumed that each cell fires independently and in accordance with a Poisson distribution, simplifying the likelihood expression to:

$$\underset{x}{\operatorname{argmax}} \, [P(\overrightarrow{pv}|x)] = \underset{x}{\operatorname{argmax}} \left[\prod_{i=1}^{n} p(r_i|x)\right] = \underset{x}{\operatorname{argmax}} \left[\prod_{i=1}^{n} \frac{\lambda_i(x)^{r_i}}{r_i!} \, e^{-\lambda_i(x)}\right]$$

where *i* is the place cell index, r_i is the firing rate of place cell *i* for a given time bin, and $\lambda_i(x)$ is the mean firing rate of place cell *i* at spatial location x. To further simplify the expression, we chose to estimate the loglikelihood instead of just the likelihood, resulting in the expression:

$$\underset{x}{\text{argmax}}\,[\ln P(\overrightarrow{pv}|x)]$$

$$= \underset{x}{\text{argmax}}\left[\sum_{i=1}^{n} r_i \ln \lambda_i(x) - \lambda_i(x) - \ln\,(r_i!)\right] = \underset{x}{\text{argmax}}\left[\sum_{i=1}^{n} r_i \ln \lambda_i(x) - \lambda_i(x)\right]$$

where the term $-\,ln(r_i!)$ could be ignored since r_i is constant across all classes x.

To run the decoder the data was split into training and testing frames. When training and testing within the same session (that is, train and test within environment A), odd time bins were used to train and even time bins to test the decoder. When decoding across sessions (decoding position in A' or B based on the activity in A), all time bins were used. Time bin sizes where set to 3 frames (0.4 seconds) and the deconvolved calcium signal was summed across these three frames to get the neural signal. The open field box was split into 32 × 32 spatial bins x. Training data was used to calculate spatial tuning maps, from which $\lambda_i(x)$ in the decoder was estimated. The Euclidian distance between the decoded position, given by the peak in the likelihood matrix per time bin, and the actual position of the animal was defined as the decoding error. Only time bins in which the animal was moving at least 2.5 cm/s were used to calculate the decoding error.

When comparing the decoding error from anatomically close place cells and random place cells, we performed decoding from all sessions and both environments A and B (n = 24 sessions). We trained and tested the decoder on the same session and split the data in odd and even time bins as described above for training and testing. For each session, a unique reference place cell was picked at random, and decoding was performed using all anatomical neighbouring cells within an incremental distance. As a randomly chosen control for each cell at each anatomical distance (five in total), decoding was performed using a size-matched sample of randomly picked place cells from the same session. From each session, we randomly picked 200 reference cells, and performed 25 randomly picked controls for each distance for each cell.

Classification of object-tuned cells

Object-tuned cells were classified based on procedures in previous reports of object-tuned cells in mice (*15, 16*) but adapted to calcium imaging data. We considered fields at all locations from the object, not limiting the search to predefined templates of distances from and direction to the object as in previous work (*17*). Only cells with SNR > 3 and at least 100 calcium events in total over all four trials were considered. To be classified as object-tuned, cells had to fulfil all three of the following criteria:

1) Cells needed to have at least one new firing field emerge from the first “Open field” trial to the “Object” trial. Any field centroid in “Object” had to be at least 15 cm away from any other field centroid within “Open field” to be considered a unique, new field.
2) Cells needed to have a substantial spatial information (SI) value in “Object”. The SI of the cell was compared to the SI of a shuffled distribution of its calcium events. Each cell’s calcium events were circularly shuffled for 200 iterations, and for each shuffle a tuning map and the SI was calculated. The recorded SI of the cell had to exceed the 99th percentile of the shuffled distribution.
3) The cells also had to display stability between the two object trials. To quantify this, we calculated an “Object Vector score” (OV score), as described by others (*15, 16*). The OV score is defined as the Pearson’s correlation between the allocentric tuning maps (see *Spatial tuning maps and allocentric tuning maps*) of “Object” and “Object moved” (see Fig. 8B). A shuffled distribution of OV scores was created by circularly shuffling the cells activity in both “Object”

and “Object moved” 200 times and recalculating the OV score for each pair. A cell passed the OV score criterion if it surpassed the *99th* percentile of the shuffles.

Statistical tests

All statistical tests were two-sided unless otherwise specified. The tests used with their results are noted within the figure legends or directly within the figures if not within the main text. Most statistical tests were performed using the Python packages SciPy and AstroPy. The calculation of mutual information for continuous variables was performed using the Python package sklearn.

Data and code availability

All code used for data analysis is available at GitHub (https://github.com/torsteinsl/ca1topography). The data is made available via DOI (https://doi.org/10.25493/TRTW-NK8).

References

1. T.-W. Chen *et al.*, Ultrasensitive fluorescent proteins for imaging neuronal activity. *Nature* **499**, 295–300 (2013).
2. H. Dana *et al.*, Thy1-GCaMP6 transgenic mice for neuronal population imaging in vivo. *PLoS One* **9**, e108697 (2014).
3. A. F. Au - Ulivi *et al.*, Longitudinal Two-Photon Imaging of Dorsal Hippocampal CA1 in Live Mice. *JoVE*, e59598 (2019).
4. M. G. Velasco, M. J. Levene, In vivo two-photon microscopy of the hippocampus using glass plugs. *Biomed Opt Express* **5**, 1700–1708 (2014).
5. W. Zong *et al.*, Large-scale two-photon calcium imaging in freely moving mice. *Cell* **185**, 1240–1256.e1230 (2022).
6. A. Mathis *et al.*, DeepLabCut: markerless pose estimation of user-defined body parts with deep learning. *Nat. Neurosci.* **21**, 1281–1289 (2018).
7. M. Pachitariu *et al.*, Suite2p: beyond 10,000 neurons with standard two-photon microscopy. *bioRxiv*, 061507 (2017).
8. M. Pachitariu, C. Stringer, K. D. Harris, Robustness of spike deconvolution for neuronal calcium imaging. *The Journal of Neuroscience*, 3339–3317 (2018).
9. D. M. Grijseels, K. Shaw, C. Barry, C. N. Hall, Choice of method of place cell classification determines the population of cells identified. *PLoS Comput. Biol.* **17**, e1008835 (2021).
10. W. E. Skaggs, B. L. McNaughton, M. A. Wilson, C. A. Barnes, Theta phase precession in hippocampal neuronal populations and the compression of temporal sequences. *Hippocampus* **6**, 149–172 (1996).
11. P. A. P. Moran, Notes on Continuous Stochastic Phenomena. *Biometrika* **37**, 17–23 (1950).
12. H. A. Obenhaus *et al.*, Functional network topography of the medial entorhinal cortex. *Proceedings of the National Academy of Sciences* **119**, e2121655119 (2022).
13. H. S. Wirtshafter, J. F. Disterhoft, Place cells are nonrandomly clustered by field location in CA1 hippocampus. *Hippocampus* **33**, 65–84 (2023).
14. K. Zhang, I. Ginzburg, B. L. McNaughton, T. J. Sejnowski, Interpreting Neuronal Population Activity by Reconstruction: Unified Framework With Application to Hippocampal Place Cells. *J. Neurophysiol.* **79**, 1017–1044 (1998).
15. O. A. Hoydal, E. R. Skytoen, S. O. Andersson, M. B. Moser, E. I. Moser, Object-vector coding in the medial entorhinal cortex. *Nature* **568**, 400–404 (2019).
16. S. O. Andersson, E. I. Moser, M.-B. Moser, Visual stimulus features that elicit activity in object-vector cells. *Communications Biology* **4**, 1219 (2021).
17. A. Nagelhus, S. O. Andersson, S. G. Cogno, E. I. Moser, M.-B. Moser, Object-centered population coding in CA1 of the hippocampus. *Neuron* **111**, 2091–2104.e2014 (2023).

SF1

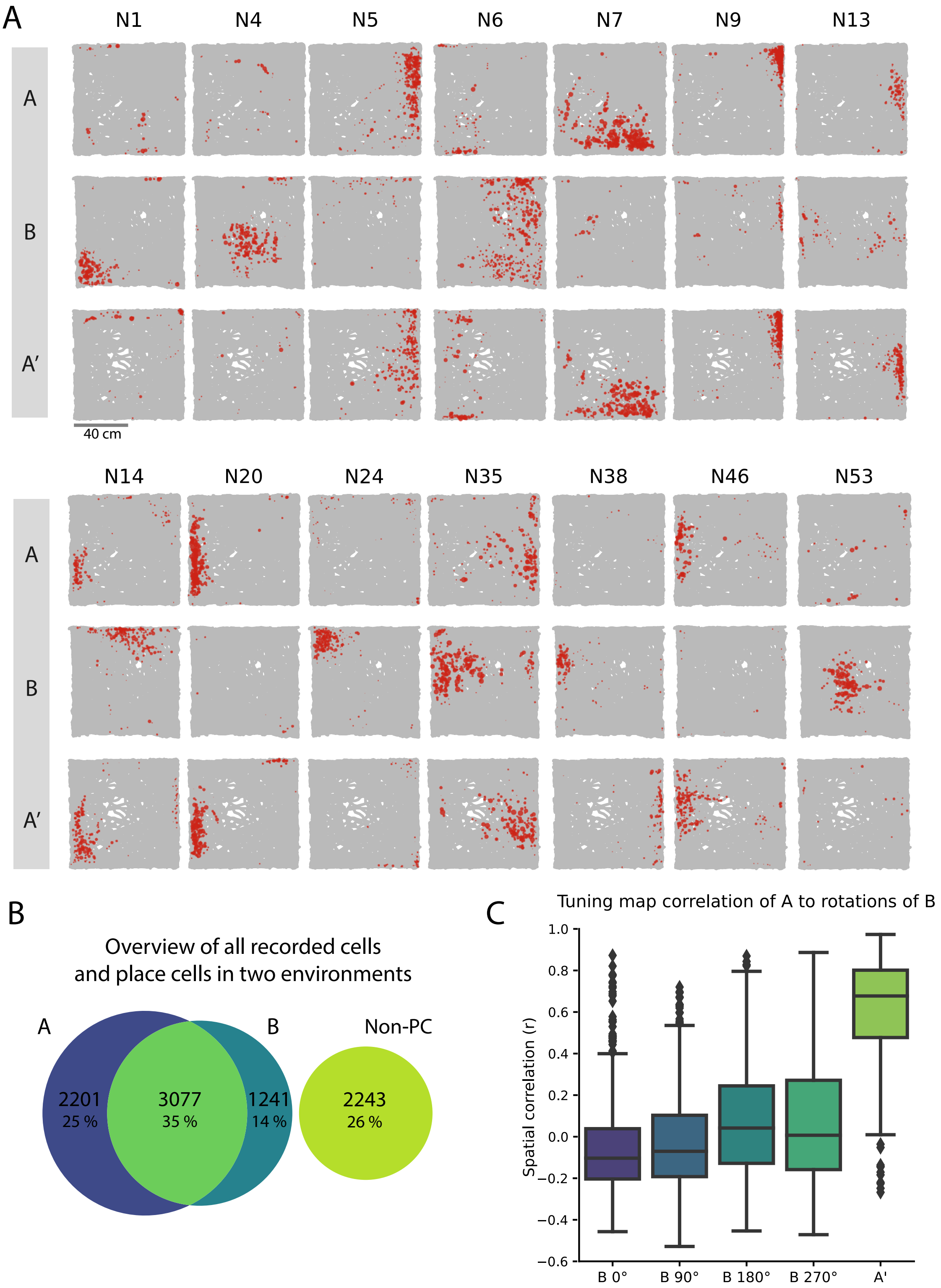
A
N1
N4
N5
N6
N7
N9
N13
A
B
A′
40 cm
N14
N20
N24
N35
N38
N46
N53
A
B
A′
B
Overview of all recorded cells
and place cells in two environments
A
B
Non-PC
2201
25 %
3077
35 %
1241
14 %
2243
26 %
C
Tuning map correlation of A to rotations of B
Spatial correlation (r)
1.0
0.8
0.6
0.4
0.2
0.0
−0.2
−0.4
−0.6
B 0°
B 90°
B 180°
B 270°
A'

Supplementary figure 1: 2P imaging of place cells in CA1

- **A. Example place cells**. From an example session with 751 place cells, the trajectory maps for 14 place cells are shown (columns) on successive tests in environments A and B (A-B-A' sequence) (rows). The running trajectory of the mouse is plotted in grey; deconvolved calcium events are superimposed as red dots (size indicates amplitude of the deconvolved signal).
- **B. Number and percentage of place cells in each environment**. Venn diagram showing the distribution of place cells and non-place cells (non-PC) in mouse CA1 across environments for all five mice (group A consists of place cells in either one or both of the recordings in environment A).
- **C. Rotations of the environment do not explain remapping.** Box plot (symbols as in **Fig. 3D**) showing the correlation between the spatial tuning maps of place cells in environment A (n = 495 place cells) and the spatial tuning maps of the same cells in environment B and A' from one example session. High correlations from A to A' (*median correlation of A-A' with 99 % CI from bootstrapping with 10,000 resamples: 0.86 [0.64, 0.70])* were not matched by similar correlations between A and B (median = -0.10), and rotation of environment B did not change the correlation (B 90°: median = -0.070; B 180°: median = 0.042; B 270°: median = 0.0068. The low correlation in spatial tuning between different environments (A to B) is not caused by rotation of the spatial tuning maps between environments. An orthogonal spatial representation is maintained between environments, consistent with global remapping.

SF2

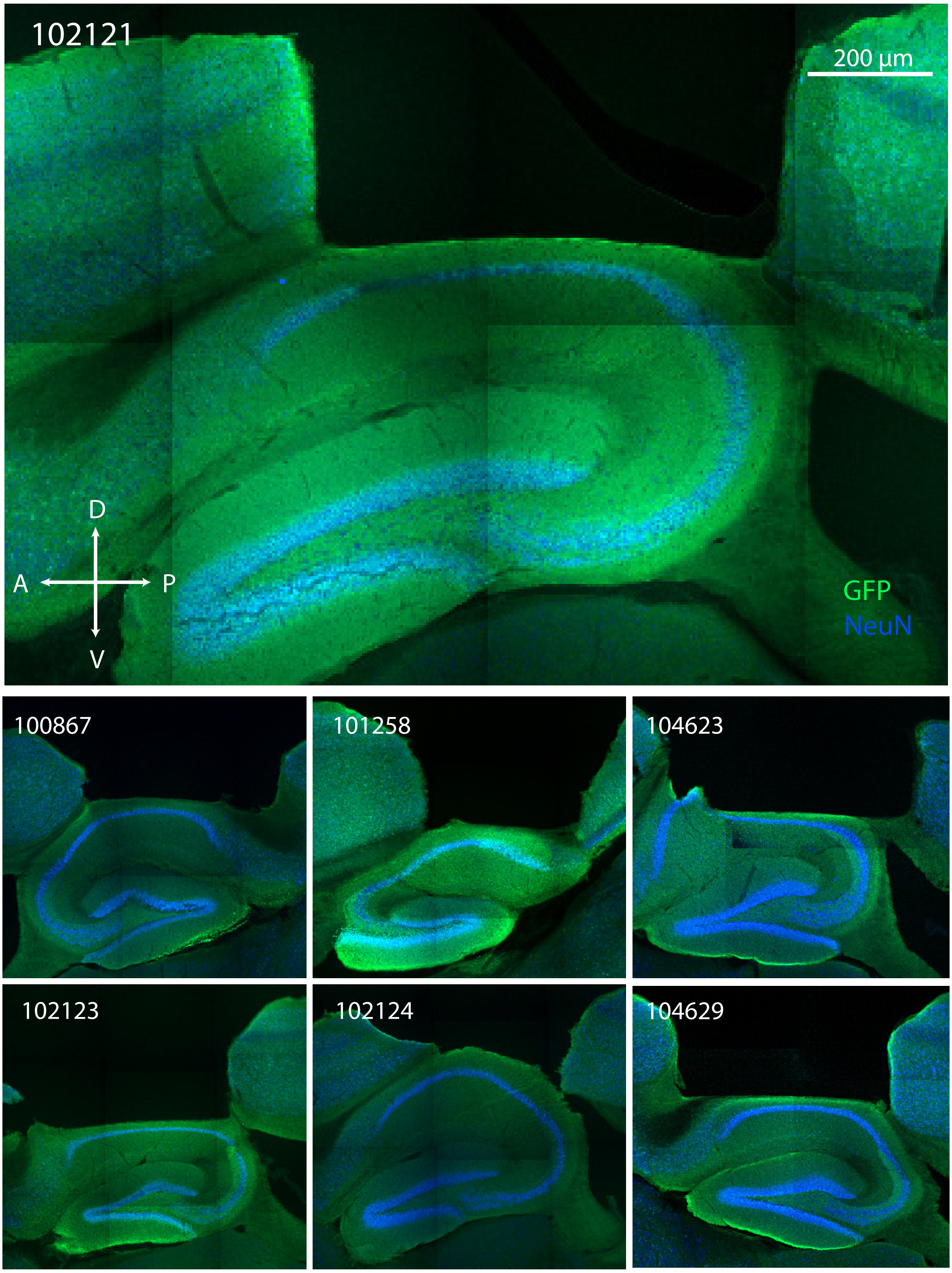
102121
200 µm
D
A
P
V
GFP
NeuN
100867
101258
104623
102123
102124
104629

Supplementary figure 2: Histology from all seven animals showing aspiration of overlying cortex with site of glass plug implant directly above dorsal CA1

Epifluorescent images showing sagittal sections immunostained for GFP (green) and NeuN (blue) for all animals. Above the CA1, the area of aspirated cortex and implantation site for our glass plug is clearly visible, allowing widespan access to image the pyramidal layer of dorsal CA1. Animal ID, scale bar, anatomical axes (A: anterior, P: posterior, D: dorsal, V: ventral) and colour scheme are as indicated.

SF3

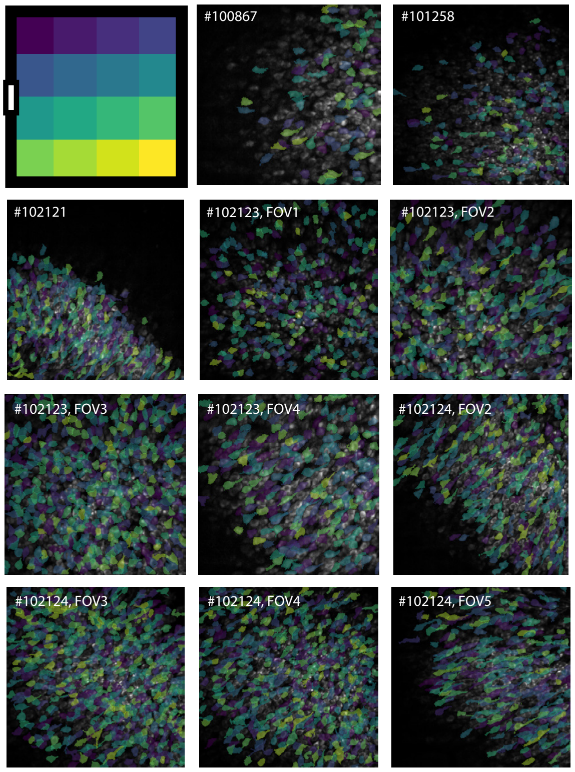
#100867
#101258
#102121
#102123, FOV1
#102123, FOV2
#102123, FOV3
#102123, FOV4
#102124, FOV2
#102124, FOV3
#102124, FOV4
#102124, FOV5

Supplementary figure 3: Place field locations of CA1 place cells are scattered across the FOV

As in **Fig. 2A**, the top left panel shows environment A split into 16 sections with a unique colour. All FOVs used in the following analyses are shown in separate panels (12 sessions in total, including **Fig. 2A**). For all cells that were classified as place cells in environment A, the location of their place field in environment A is indicated by colour-coding the cells with reference to the colour matrix of environment A.

SF4

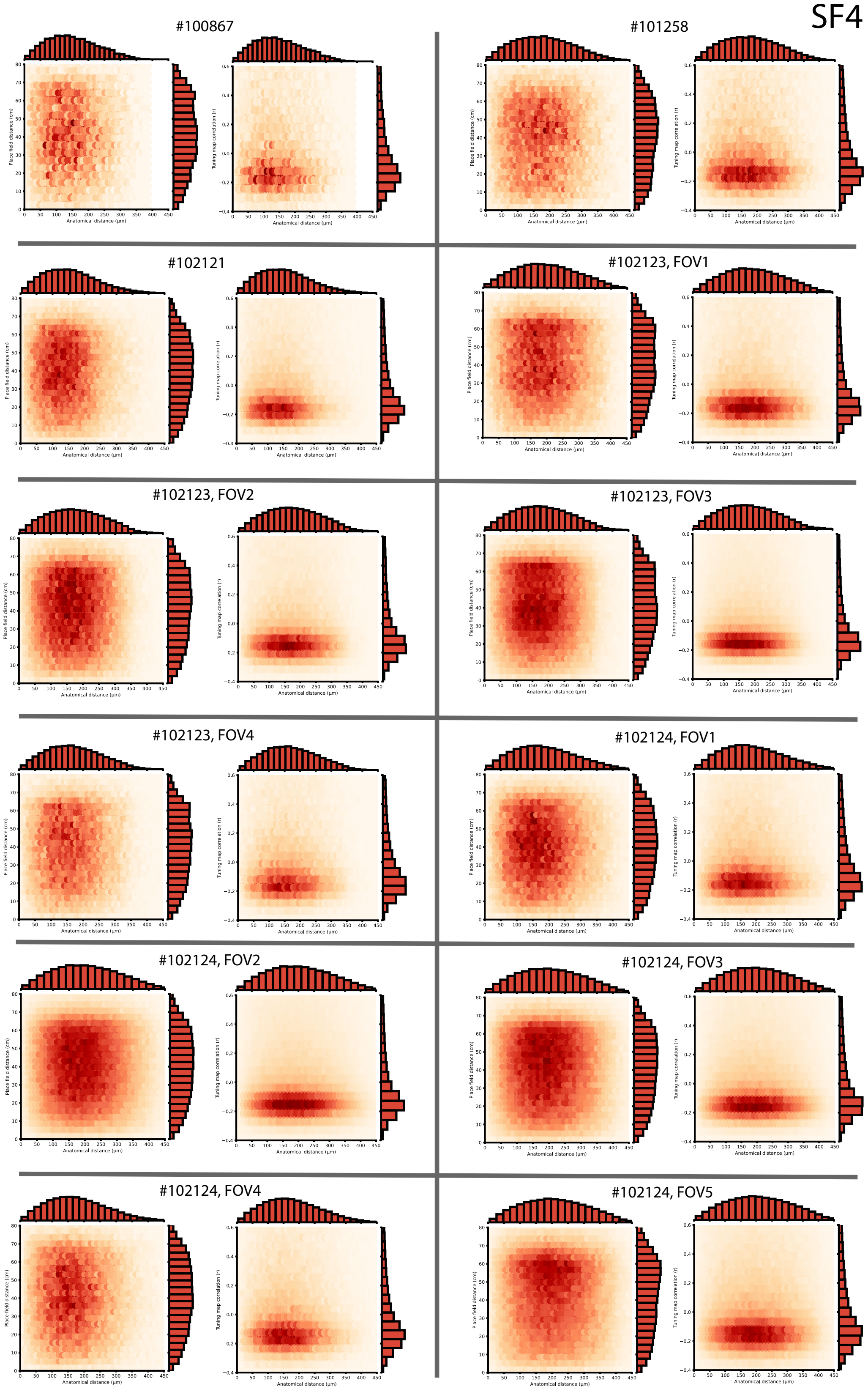

#100867
#101258
#102121
#102123, FOV1
#102123, FOV2
#102123, FOV3
#102123, FOV4
#102124, FOV1
#102124, FOV2
#102124, FOV3
#102124, FOV4
#102124, FOV5
Place field distance (cm)
Tuning map correlation (r)
Anatomical distance (μm)

Supplementary figure 4: Comparison of anatomical distance and spatial properties for place cell pairs in single sessions

Pairwise analyses are shown for each single session (n = 12 sessions). As in **Fig. 2BC**, showing the anatomical distance between a place cell pair compared to the place field distance (left in each subsection) and the correlation of spatial tuning maps (right in each subsection). As in the pooled data in **Fig. 2BC**, we could not identify a population of cell pairs with both short anatomical distances and short place field distances, or both short anatomical distance and high correlation of their spatial tuning maps. The tendency of slightly negative correlations are apparent across experiments, and are likely a result of most place cells not sharing location of their place field within the environment resulting in slightly negative correlations.

SF5

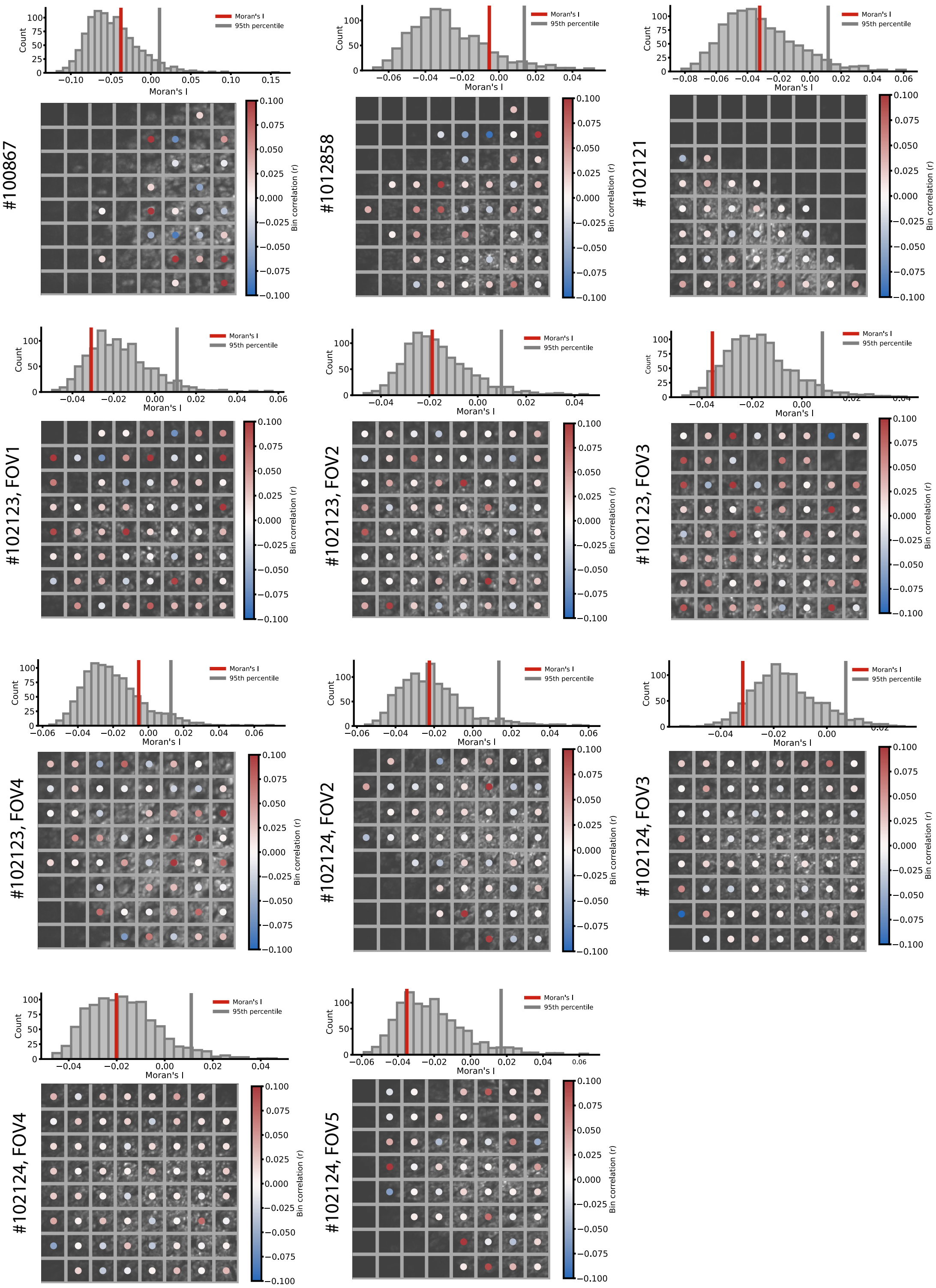
#100867
#1012858
#102121
#102123, FOV1
#102123, FOV2
#102123, FOV3
#102123, FOV4
#102124, FOV2
#102124, FOV3
#102124, FOV4
#102124, FOV5
Moran's I
95th percentile
Count
Bin correlation (r)

Supplementary figure 5: Analyses in individual animals and FOVs reveal no clustering of spatially correlated place cells

To assess if there are clusters of similarly correlated anatomical bins within the FOVs, global Moran's I was calculated for each single session (separately for both environment A and B, n = 24), as presented in **Fig. 3E**. Here the results from the remaining 11 sessions for environment A are shown (data for 12 sessions from environment B are not shown; results mirrored those in environment A). As in **Fig. 3E**, the correlations within each anatomical bin are close to 0. When calculating the global Moran's I for each FOV, the calculated I fell well within a randomly picked control distribution for all sessions, noticeable in the histograms above each FOV (Moran's I < 95th percentile, red vertical line is lower than the grey vertical line). Noticeably, all values of global Moran's I are negative yet close to 0, and thus tend to match the expected value of I (see Methods for details), consistent with a random distribution of values across the anatomical bins, with no signs of clustering.

SF6

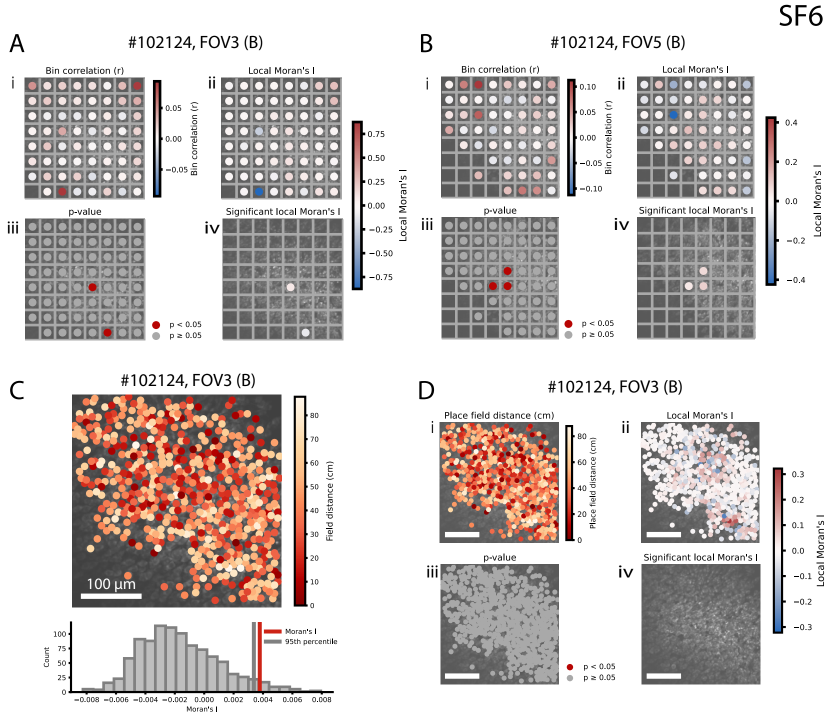
A
#102124, FOV3 (B)
i
Bin correlation (r)
ii
Local Moran's I
iii
p-value
iv
Significant local Moran's I
p < 0.05
p ≥ 0.05
B
#102124, FOV5 (B)
C
#102124, FOV3 (B)
100 µm
Field distance (cm)
Moran's I
95th percentile
Count
D
#102124, FOV3 (B)
Place field distance (cm)

Supplementary figure 6: Local Moran's I reveal no clusters of high-correlated anatomical bins

To complement analyses of global Moran's I, we determined the local Moran's I of all anatomical bins to identify any localised clusters not revealed by global Moran's I (for details, see Methods). For all trials (12 sessions with environment A and B, n = 24), we used the correlation within anatomical bins (i, correlation value as indicated by colour bar to the right) and calculated the local Moran's I per bin (ii, I-values as indicated by colour bar to the right). We then calculated the p-value per bin (iii) and defined significantly extreme values of local Moran's I by a level of $p < 0.05$ (significant bins are coloured in red). We then assessed the local Moran's I for the significant bins (iv). For all sessions, only two had anatomical bins with significant local Moran's I, showed in panel A and B, respectively.

- **A.** One of the two sessions containing anatomical bins with significant local Moran's I. For this session, two lone standing bins were significant, one having a low positive bin correlation (0.020) and the other a low negative bin correlation (-0.0022). Local Moran's I values for these bins were near 0 (-0.11 and 0.084, respectively).
- **B.** Similar to panel A, for the second session with anatomical bins with significant local Moran's I, showing a neighbouring cluster of three significant anatomical bins. These three bins have a bin correlation close to 0 (0.0016, 0.0076 and -0. 0019) and low slightly positive local Moran's I values (0.071, 0.043 and 0.093). This indicates a small cluster of non-correlated anatomical bins and not a cluster of similarly tuned place cells.
- **C.** In a complementary, bin-free approach we used the cells' location rather than the bins, and calculated the place field distance from each single place cell to that of its most nearby neighbouring place cell. In 1/24 sessions, the global Moran's I was higher than the 95th percentile of a randomly picked distribution ($p = 0.043$) and is depicted here. Top: FOV with place cells indicated by superimposed dots coloured by the place field distance to its nearest neighbour from the significant session (scale bar at bottom left). Bottom: Histogram showing the random distribution of Moran's I for this session with the 95th percentile of the random distribution (grey vertical line) and the calculated Moran's I (red vertical line).
- **D.** Decomposing the global Moran's I in **C** into local Moran's I, as in **A**-**B**, gave no significant cells or clusters indicating the existence of similarly tuned nearby neighbouring place cells. Scale bar (100 µm) at bottom left.

SF7

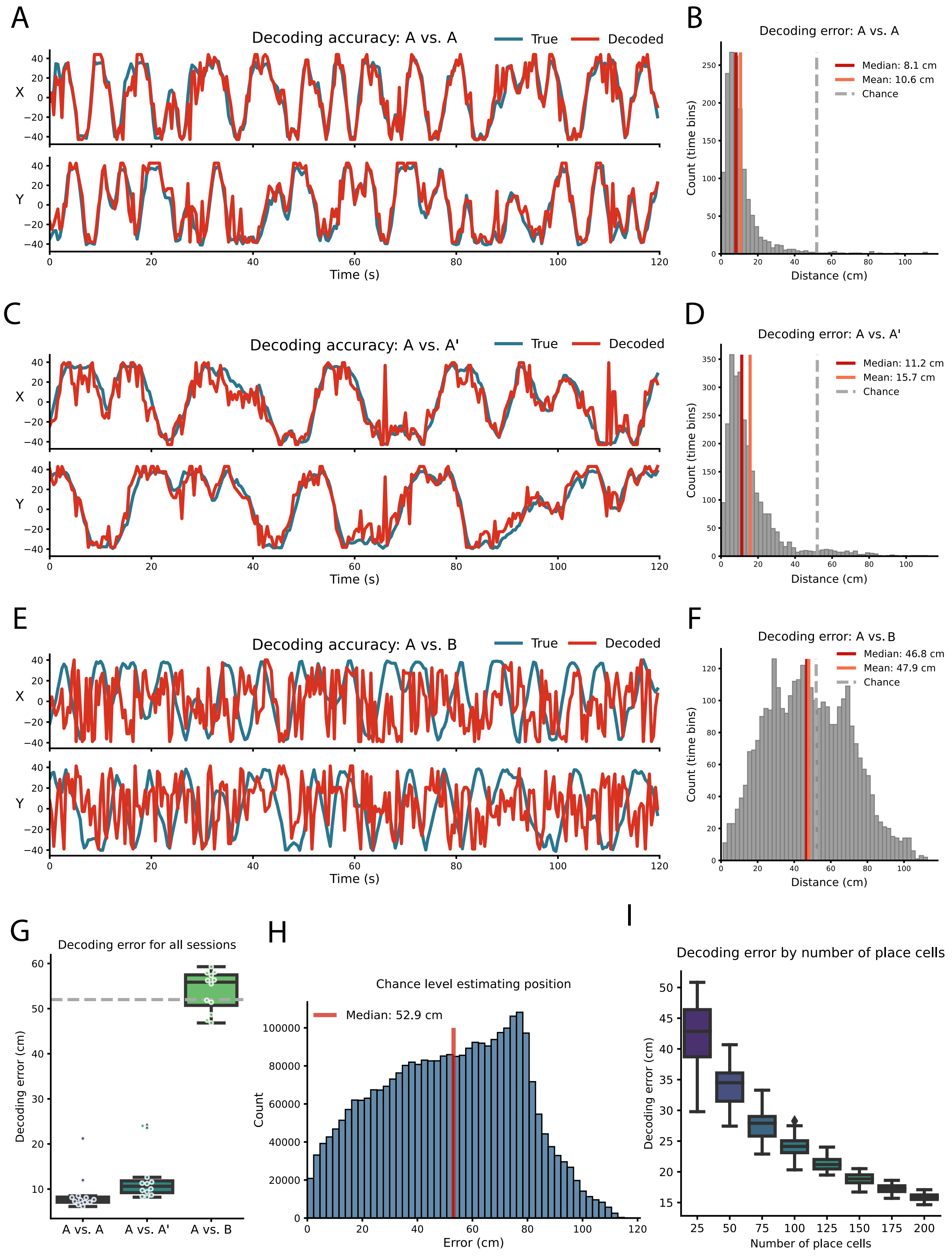
A
Decoding accuracy: A vs. A
True
Decoded
X
Y
Time (s)
B
Decoding error: A vs. A
Median: 8.1 cm
Mean: 10.6 cm
Chance
Count (time bins)
Distance (cm)
C
Decoding accuracy: A vs. A'
True
Decoded
X
Y
Time (s)
D
Decoding error: A vs. A'
Median: 11.2 cm
Mean: 15.7 cm
Chance
Count (time bins)
Distance (cm)
E
Decoding accuracy: A vs. B
True
Decoded
X
Y
Time (s)
F
Decoding error: A vs. B
Median: 46.8 cm
Mean: 47.9 cm
Chance
Count (time bins)
Distance (cm)
G
Decoding error for all sessions
Decoding error (cm)
A vs. A
A vs. A'
A vs. B
H
Chance level estimating position
Median: 52.9 cm
Count
Error (cm)
I
Decoding error by number of place cells
Decoding error (cm)
Number of place cells

Supplementary figure 7: Performance of Bayesian decoder of position from CA1 place cells

A. **Accuracy of positional decoding from CA1 place cells.** Position was decoded using a Bayesian decoder trained and tested on all place cells in this session. The session was binned to 0.4-second bins (three frames per bin). Odd bins were used to train the decoder while even bins were used to test its performance. For X (top) and Y (bottom) positions in space, the true position (blue line) was compared to that of the decoded position (red line).
B. **Decoding error from training and testing in box A**. For the data in **A**, the decoding error was determined as the Euclidean distance from the decoded position to the actual position of the animal per time bin. Mean and median decoding error is denoted in the figure, together with chance level (panel **H**).
C. **Position can be decoded in box A' based on activity in box A.** Like in figure **A**, the figure shows the decoding accuracy for X and Y positions of the animal when the decoder was trained on all place cells in the entire session A and tested on the entire session A'.
D. **Decoding error from training in box A and testing in box A' is similar to testing in box A.** As in panel **B**, the decoding error from session A to A' is comparable to that of session A-A.
E. **Position is poorly decoded in box B when the decoder is trained in box A.** Comparable to **A** and **C**, the decoding accuracy for X and Y position when training on all place cells in the entire session A and decoding on the entire session B. The performance is clearly worse than that of A-A or A-A'.
F. **Decoding error in box B when trained in box A is comparable to chance level.** As in **B** and **D**, showing the decoding error from session A-B. Consistent with the results in **E**, the decoding error is clearly different from that of A-A and A-A', and centres around chance levels. The results are consistent with an orthogonal representation of space by CA1 place cells after remapping.
G. **Decoding error for all sessions.** Box plot showing the decoding error between environments for all sessions, superimposed with dots (one per session) for each single datapoint (n = 12 sessions). All place cells were used to decode. The decoding error drastically increases across environments (A to B, median decoding error with 99 % CI from bootstrapping with 10,000 resamples: 55.86 cm [48.65, 57.91]) compared to within environments (A to A, median = 7.62 cm; and A to A', median = 10.62 cm), and matches chance levels across environments.
H. **Determining chance level.** Histogram showing the position offset (error) after circular shuffling of the animals' position and subsequent calculation of Euclidean distance from true position to the shuffled position at each timepoint. The shuffle was performed 200 times for each session, resulting in a chance decoding error of 52.9 cm. The median was chosen due to skewedness of distribution.
I. **Decoding performance increases with number of place cells**. Box plot showing decoding error as a function of how many place cells used to perform the decoding. As expected, the performance of the decoder increases gradually as the number of place cells used to decode increases.

SF8

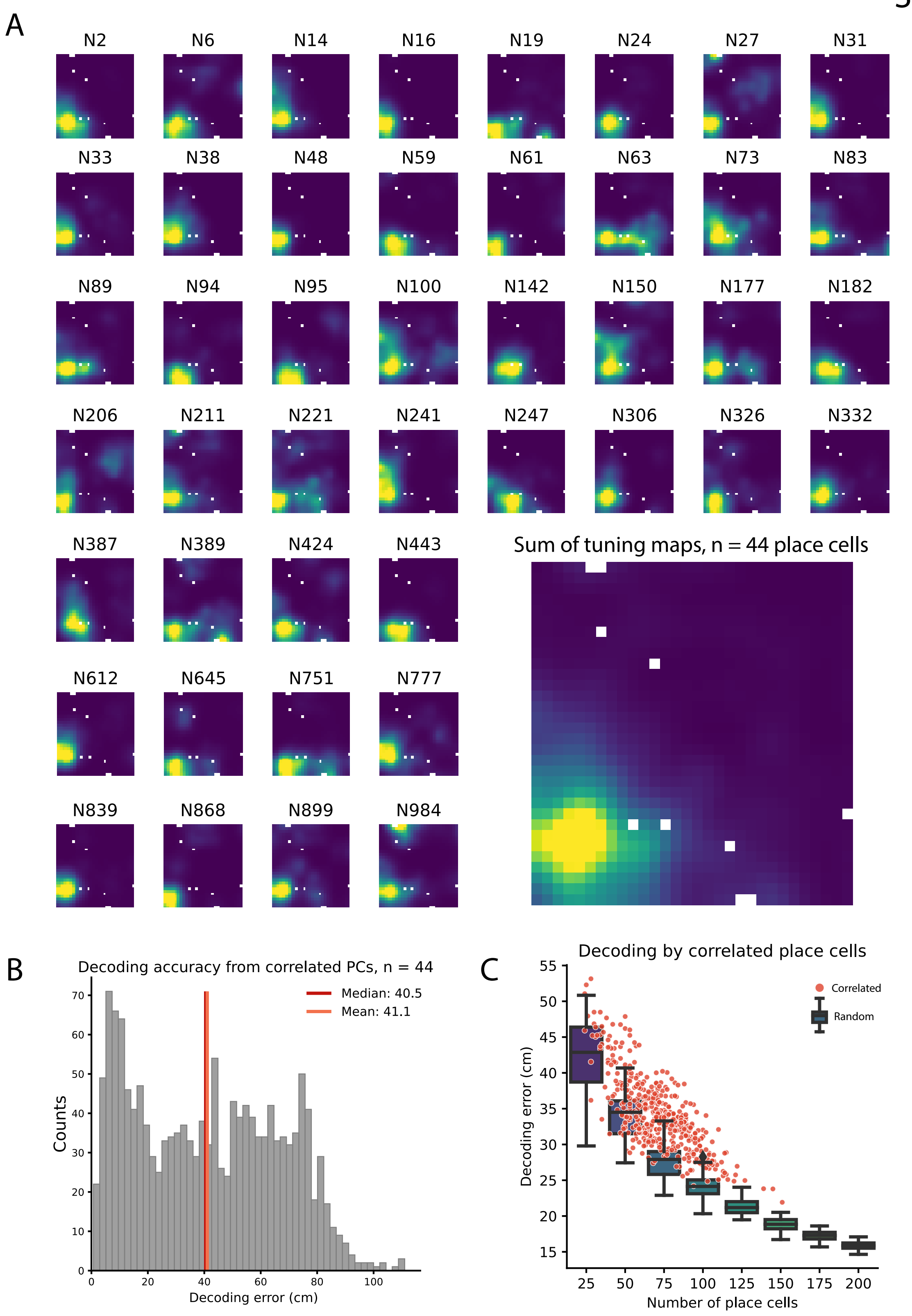

A
N2
N6
N14
N16
N19
N24
N27
N31
N33
N38
N48
N59
N61
N63
N73
N83
N89
N94
N95
N100
N142
N150
N177
N182
N206
N211
N221
N241
N247
N306
N326
N332
N387
N389
N424
N443
Sum of tuning maps, n = 44 place cells
N612
N645
N751
N777
N839
N868
N899
N984
B
Decoding accuracy from correlated PCs, n = 44
Median: 40.5
Mean: 41.1
Counts
Decoding error (cm)
C
Decoding by correlated place cells
Correlated
Random
Decoding error (cm)
Number of place cells

Supplementary figure 8: Decoding from correlated place cells

A. **Spatial tuning maps of highly correlated place cells**. To see the effect of decoding from only similarly tuned place cells, we first picked one 'reference place cell'. In this example, we used 'N2' as our reference. We then correlated the spatial tuning map of our reference place cell to that of all other place cells. The plot shows the 43 highest correlated cells to place cells N2. To highlight the spatial tuning of these 44 place cells, the sum of all spatial tuning maps is plotted in the bottom right corner, showing a clear preference for one corner for these place cells.

B. **Performance of decoding from correlated place cells**. Decoding error, measured as the Euclidean distance between the true position of the animal and the decoded position, when decoding from these 44 correlated place cells results in a decoding error of 40.5 cm (figure as in **Supplementary Fig. 7BDF**). The decoding error appears better than chance (53 cm, see **Supplementary Fig. 7H**), as expected since parts of the environment (lower left corner) is well represented in the training data.

~~**C.**~~ **Decoding from correlated place cells performs worse than for randomly picked place cells**. Box plot represent decoding error as a function of number of randomly picked place cells used to decode, exactly as in **Supplementary Fig. 7I**. Superimposed as red dots is the decoding error as a function of cell number for different cell samples consisting of place cells with a spatial tuning map correlation > 0.4 (n = 495 place cell samples). The samples were determined by randomly picking a reference place cell and adding the place cells with a spatial tuning map correlation of > 0.4 compared to the reference place cell. Due to low general performance with few cells, instances with 15 or less place cells to decode with were omitted. As for randomly selected place cells, the decoding error is reduced when the number of cells increases. However, the decoding error for correlated place cells is higher than from the randomly chosen place cells. For statistical comparison, the correlated place cells were binned to ± 7 for the number of place cells used to decode. For all instances, the decoding error by the correlated place cells were higher than for randomly picked place cells, and the median of the randomly picked place cells fell outside the 99 % CI of the correlated place cells (see Supplementary Table 2).

SF9

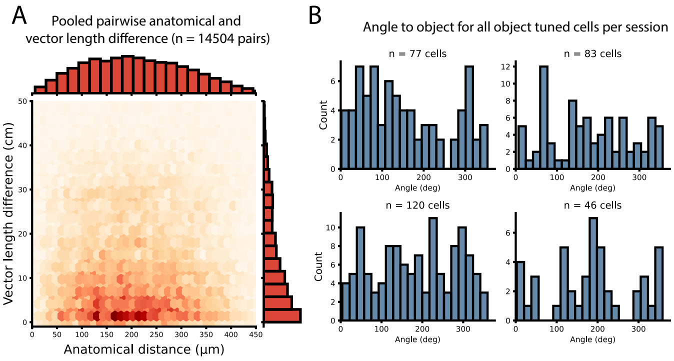
A
Pooled pairwise anatomical and
vector length difference (n = 14504 pairs)
Vector length difference (cm)
Anatomical distance (µm)
B
Angle to object for all object tuned cells per session
n = 77 cells
n = 83 cells
n = 120 cells
n = 46 cells
Count
Angle (deg)

Supplementary figure 9: Object-tuned CA1 cells are not anatomically clustered by distance from the object

A. **Field distance from object and anatomical distance are not correlated in pairs of object-tuned CA1 cells.** Heatmap similar to **Fig. 8E** for all cells in four object experiments, showing the pairwise difference in Euclidean distance from the centre of the object field to the object as a function of anatomical distance for pairs of object-tuned cells. As most object fields tend to be near the object (see **Fig. 8C**), differences in field distances from the object are generally low, mostly < 10 cm. However, their anatomical location remains distributed and were considered statistically independent (mutual information for continuous variables: MI = 0.0018).
B. **Distribution plots showing angular tuning of all object-tuned cells per experiment.** Histograms showing the angular tuning of object-tuned cells for the four experiments. There are no apparent clusters of preferred angle to object. Number of cells per experiment is indicated above the histograms.

Median decoding error (cm) with 99% CI and random controls

Anatomical distance	**Median [99% CI]**	**Median of random control**
25 μm	53.51 [52.67, 54.45]	52.90
50 μm	46.82 [46.30, 47.43]	46.31
75 μm	36.25 [35.97, 36.49]	36.04
100 μm	24.95 [24.71, 26.21]	26.00
125 μm	19.08 [18.71, 19.41]	18.92

Supplementary Table 1: Statistical testing for Figure 7A.
At each incremental anatomical distance, the decoding accuracies were similar for neighbouring place cells to that of size-matched randomly picked place cell controls. Confidence intervals were calculated by bootstrapping for 10,000 resamples. At each distance, the median decoding error of the randomly picked control fell within the confidence intervals of the neighbouring place cells.

Median decoding error (cm) with 99% CI from correlated and random place cells

Number of place cells	**Median [99% CI]**	**Median of random place cells**
25	46.63 [43.38, 51.07]	42.87
50	37.18 [34.93, 38.52]	34.50
75	34.03 [33.24, 34.42]	27.91
100	28.18 [27.70, 29.27]	24.13
125	25.36 [24.63, 26.95]	21.18
150	22.85 [21.92, 23.79]	18.85

Supplementary Table 2: Statistical testing for Supplementary Figure 8C. Decoding error (cm) as a function of number of place cells used to decode for correlated and randomly picked place cells. The correlated place cells were binned at ± 7 cells. The median decoding error and the 99% CI from bootstraps with 10,000 resamples for the correlated place cells indicate a worse decoding than from randomly picked place cells.
